# Associations between skin bacteria and chytrid fungal infection in Asian amphibians

**DOI:** 10.1016/j.isci.2025.113661

**Published:** 2025-09-29

**Authors:** Jiaqi Zhang, Xuejiao Yang, Xavier A. Harrison, Shaofei Yan, Xianglei Hou, Supen Wang, Cunxia Xu, Teng Deng, Tianjian Song, Mingshuo Qin, Xuan Liu, Trenton W.J. Garner, Matthew C. Fisher, Yiming Li

**Affiliations:** 1 College of Life Science, Ningxia University, 539 Helanshan West Road, Xixia District, Yinchuan, Ningxia, China; 2 Key Laboratory of Animal Ecology and Conservation Biology, Institute of Zoology, Chinese Academy of Sciences, 1 Beichen West Road, Chaoyang, Beijing 100101, China; 3 University of Chinese Academy of Sciences, Beijing 100049, China; 4 Institute of Zoology, Zoological Society of London, Regent’s Park, London NW1 4RY, UK; 5 College of Life Science, Anhui Normal University, Wuhu, Anhui 241000, China; 6 Department of Infectious Disease Epidemiology, Imperial College, School of Public Health, 80 Wood Lane, London W2 1PG, UK; 7 School of Life Sciences, Hebei University, Baoding 071002, China

**Keywords:** Ecology, Zoology, Microbiology

## Abstract

Identifying the generality of defensive symbionts and microbiome structures associated with pathogen infection across multiple hosts provides insights into understanding disease susceptibility and managing disease. The fungus *Batrachochytrium dendrobatidis* (*Bd*) infects amphibian skin (the disease, chytridiomycosis), causing amphibian declines worldwide except in Asia. Here, we investigated associations between amphibian skin bacterial microbiome and *Bd* infection in five Asian amphibian species and the susceptible, Australasian species *Litoria caerulea* via experiments and in the wild. We found that Asian amphibians showed resistance to *Bd* infection experimentally, with *Bd* infection causing divergence in microbiome structures in inoculated animals. Alpha diversity and relative abundances of 16 OTUs had negative effects on *Bd* load across inoculated animals. Compared with L. *caerulea*, four OTUs (Bradyrhizobium, Achromobacter, Sediminibacterium, and Rhodoplanes) with negative effects on *Bd* load were consistently enriched in experimental and wild populations of Asian amphibians. These OTUs are probably associated with reduced *Bd* loads in Asian amphibians.

## Highlights

• *Batrachochytrium dendrobatidis* (*Bd*) infects amphibian skin, causing their decline except in Asia
• Asian amphibians showed resistance to the fungal *Bd* infection in laboratory experiments
• α diversity and 16 OTUs of the skin bacterial microbiome were negatively associated with *Bd* load
• Four OTUs likely provided protection against the *Bd* infection in Asian amphibians

## Graphical abstract

**Figure:**
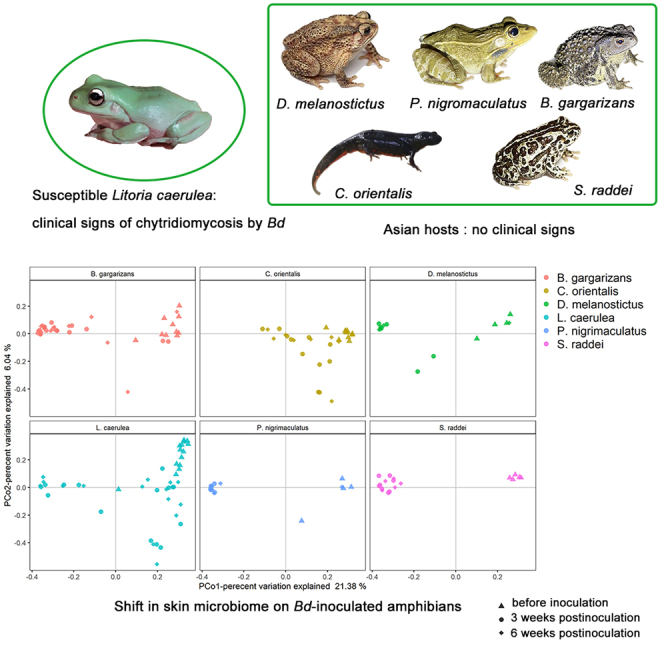

## Introduction

Multiple organisms harbor complex symbiotic microbial communities (microbiomes), which affect host health, development, behavior and disease susceptibility.^1^^,^^2^^,^^3^ Defensive symbionts protect their hosts from pathogens through mechanisms such as competition for resources,^4^ antibiotic production,^5^ or enhancing the innate immune function of the host.^6^ While microbial community structure is linked to host immune resistance against pathogens,^7^^,^^8^ pathogen invasion can also alter the diversity and structure of the microbial community.^1^^,^^9^^,^^10^ Most pathogens, if not all, infect multiple hosts spanning different species, even different orders,^11^ which are likely to show markedly different severities of host disease (from individual death to no clinical signs of disease). However, we have limited knowledge on the generality of defensive symbionts and microbial community structures that are associated with pathogen infection across multiple hosts.^2^

The fungus *Batrachochytrium dendrobatidis* (*Bd*) infects the cutaneous tissues of amphibians and causes chytridiomycosis, which has been implicated in hundreds of amphibian declines and extinctions.^12^ Declines and extinctions have been reported in many regions across the world,^12^^,^^13^ including Australia, North, Central, and South America, and Europe. Studies have shown that cutaneous symbiotic bacteria contribute to protecting amphibian hosts against *Bd* infection and reducing disease susceptibility.^14^^,^^15^^,^^16^ *In vitro*, a range of bacterial symbionts isolated from amphibians can inhibit the growth of *Bd*,^14^^,^^17^^,^^18^^,^^19^ possibly through antifungal compounds.^20^^,^^21^ The initial structure of symbiotic bacterial communities can be associated with the infection outcome of *Bd* exposure, and defensive symbiotic bacteria have been tested as probiotics to treat chytridiomycosis in frogs caused by *Bd*.^14^^,^^22^^,^^23^ Higher bacterial diversity and/or the stability of skin bacterial communities over time has been shown to confer greater resistance to *Bd* infection.^23^^,^^24^^,^^25^^,^^26^ While an increasing body of work has examined interactions between the amphibian skin-microbiome and *Bd*, this research has focused mainly on amphibians in regions where *Bd* is purported to be a recent invader, such as northern and central America and Europe.^23^^,^^27^^,^^28^^,^^29^^,^^30^^,^^31^ Knowledge of the symbiotic microbiome of Asian amphibians is limited.^32^^,^^33^^,^^34^^,^^35^

Asia has a high number of *Bd* haplotypes.^36^^,^^37^^,^^38^^,^^39^^,^^40^^,^^41^ Both the global pandemic lineage *Bd*GPL and endemic *Bd*Asia-3 and ancestral *Bd*Asia-1 occur in and are thought to be enzootic to Asia. The latter is proposed as the source of all batrachochytrids.^36^^,^^42^^,^^43^^,^^44^^,^^45^^,^^46^^,^^47^ Asian native amphibians generally present no clinical signs of chytridiomycosis, with several species showing sublethal symptoms.^39^^,^^48^^,^^49^ *Bd* prevalence in the wild and pet or food markets is high or low depending on the species, and the load of infection is generally low.^35^^,^^48^^,^^50^^,^^51^^,^^52^ Mortality events and declines in amphibians due to chytridiomycosis have not been reported. Several hypotheses have been developed to explain these phenomena, including Asian amphibian resistance to *Bd* infection^43^ due to long term *Bd* endemicity^42^ or unsuitable climates for *Bd* in Asia.^42^ These hypotheses, however, have rarely been examined in experiments or field conditions.^43^

Host-specific factors such as genetic background, host immunity and phylogeny,^53^^,^^54^^,^^55^ environmental factors including developmental stage, diet composition, temperature, precipitation, water pH, contaminants, captivity, microhabitats, and environmental reservoirs,^54^^,^^55^^,^^56^^,^^57^^,^^58^^,^^59^^,^^60^^,^^61^^,^^62^ and *Bd* infection intensity^9^^,^^63^ have been shown to influence the cutaneous bacterial communities of amphibians. *Bd* infection intensity and prevalence are also affected by temperature, precipitation, and host immunity.^12^^,^^64^ Under natural conditions, variation in host disease susceptibility can be difficult to identify and measure,^65^^,^^66^ and the structures of bacterial symbiont communities may differ among individuals, populations, species, and in time and space.^23^^,^^56^^,^^59^^,^^63^^,^^67^^,^^68^ Experimental approaches are therefore crucial for identifying which species are susceptible to *Bd* infection and teasing apart the interactions between hosts, *Bd* infection, skin symbiotic microbes, and environments.^9^^,^^69^

Here, we investigated the resistance of Asian amphibians to *Bd* infection and the associations between *Bd* infection and the skin bacterial microbiome via laboratory experiments and in the wild using five Asian native amphibian species and a known *Bd*-susceptible species native to Australia^43^^,^^70^^,^^71^ (*Litoria caerulea*). We experimentally inoculated these hosts with *Bd*GPL, the most prevalent *Bd* lineage in Asia.^37^^,^^38^^,^^39^^,^^40^ The native amphibians (*Bufo gargarizans*, *Duttaphrynus melanostictus*, *Strauchbufo raddei*, *Cynops orientalis*, and *Pelophylax nigromaculatus*) were chosen for the study because they represent different taxonomic groups and are widely distributed in Asia^72^ (Table S1). *B. gargarizans*, *D. melanostictus, and S. raddei* belong to Bufonidae in Anura, and *P. nigromaculatus* belongs to Ranidae, while *C. orientalis* belongs to Salamandridae in Caudata. *C. orientalis* occurs in central and eastern China,^73^ whereas the other four anuran species are distributed in many provinces of China and several Asian countries. Eggs are laid with water plants or in water by these species, and tadpoles develop aquatically. Adults of the hosts live along the edges of ponds and streams or in crop fields, but those of *C. orientalis* spend more time in water than do the anurans.^73^ Field and retrospective surveys have suggested that *B. gargarizans*, *D. melanostictus*, *S. raddei,* and *P. nigrimaculatus* exhibit a low prevalence of *Bd* infection or no infection across multiple regions from southern, eastern, southwestern, and central China.^37^^,^^45^^,^^74^ The prevalence of *Bd* for *C. orientalis* in pet markets is consistently low, and infection remains undetected in the wild.^45^ *Bd* positive individuals of these species generally show no clinical signs of chytridiomycosis.^37^^,^^45^^,^^74^ We first performed *Bd* inoculation experiments to examine differences in *Bd* infection intensity and clinical signs of the disease between Asian hosts and *L. caerulea*; We then investigated temporal changes in the skin bacterial microbiome associated with *Bd* infection intensity in the experiments. Finally, we compared the bacterial microbiome between wild populations of Asian hosts and *L. caerulea*.

## Results

### Bd inoculation experiments

We obtained postmetamorphic frogs of *B. gargarizans*, *D. melanostictus*, *S. raddei*, and *P. nigromaculatus* from the wild and purchased postmetamorphic individuals of *C. orientalis* and *L. caerulea* from pet markets in Beijing from 2015 to 2016 (STAR Methods, Table S1; Figure S1). We swabbed each individual collected, and tested the swabs for *Bd* infection via a nested PCR assay. All swabs were *Bd* negative according to the assay. We reared the animals individually in closed plastic boxes in the laboratory under conditions suitable for *Bd* growth and proliferation before the inoculation experiments (STAR Methods). We simultaneously inoculated 68 individuals, including 5 Asian hosts and *L. caerulea*, with a solution of 1x10^6^ zoospores of a *Bd*GPL isolate (STAR Methods, Table S1; Figure S1). We treated another 67 individuals with sterile water as a control group. The number of individuals was the same between the inoculated and control groups for all species except *D. melanostictus*, ranging from 8 frogs in the inoculated group and 7 in the control group in *D. melanostictus* to 15 frogs in the inoculated or control groups in *B. gargarizans* and *L. caerulea*. The experimental periods varied among hosts and lasted from 118 days for *P. nigrimaculatus* to 438 days for *B. gargarizans*, depending on infection and mortality rates. In the *Bd* inoculation groups, seven individuals of *L. caerulea* died (Figure S2A), and 4 died within 6 weeks post-inoculation. All the dead animals exhibited clinical signs of chytridiomycosis, including excess skin sloughing, appetite loss, lethargy, and a decreased righting reflex. We did not observe any deaths or clinical signs of disease affecting Asian hosts or control individuals.

We swabbed the skins of the experimental amphibians before inoculation and weekly postinoculation. We also swabbed the skin of dead *L. caerulea* within 4 h of death. We quantified the *Bd* load in the swabs via quantitative PCR (qPCR) (STAR Methods). All the animals tested were *Bd* negative for infection before exposure. All those inoculated with *Bd* tested positive at least once at 10 weeks postexposure (Table S2), whereas all the controls remained uninfected. Both dead and surviving *L. caerulea* maintained *Bd* infection over time (Table S2). The prevalence of *Bd* in each Asian host varied over time, ranging from 50 to 100%. For example, the *Bd* prevalence in *S. raddei* reached 100% in the first week and 50%, 70%, and 80% in the second, third, and fourth weeks, respectively*. Bd* infection in some individuals was too low to be detected at the second week, but was identified at the third or fourth week, resulting in a high prevalence at the third and fourth weeks.

We categorized samples of each Asian host into one group and those of *L. caerulea* into three groups for *Bd* load analysis. The three groups included *L. caerulea*, which covered all samples of 15 dead or surviving individuals during the experiments; *L. caerulea* survival, which contained samples of 8 living individuals; and *L. caerulea* dead, which covered samples of 7 dead individuals (Figure S2B). The L. *caerulea* dead group presented the strongest infection intensity at the seventh week (Figure S2B), whereas *L. caerulea* surviving and Asian amphibians generally presented weak and consistent infection intensities over the same time span. Six weeks post inoculation, *L. caerulea* had the heaviest *Bd* loads (mean log (*Bd* load+1) = 1.7957 ± 0.8559 (SD), per individual per week), followed by *S. raddei* (1.1388 ± 0.2412), *P. nigromaculatus* (1.0924 ± 0.0812), *B. gargarizans* (0.9166 ± 0.5735), *D. melanostictus* (0.6294 ± 0.2366), and *C. orientalis* (0.5165 ± 0.263). ANOVA revealed that the mean *Bd* load differed among the six host species (*p* < 0.0001) (Table S3A). Games-Howell tests revealed that *L. caerulea*, *P. nigromaculatus,* and *S. raddei* presented greater *Bd* loads than *D. melanostictus* and *C. orientalis* did. *L. caerulea* also had heavier *Bd* loads than did *B. gargarizans*. When the L. *caerulea* dead and *L. caerulea* survival groups were included in the analysis (Table S3B), the *L. caerulea* dead group presented greater *Bd* loads than the *L. caerulea* survival group did, as did each Asian host (*p* < 0.001 for all).

### Skin bacterial compositions across amphibians inoculated over time

**Figure 1:**
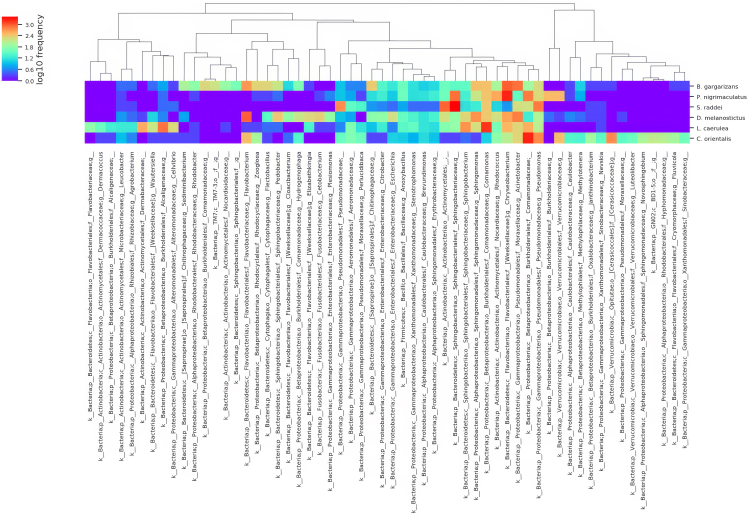
Heatmap of bacterial genera with relative abundances >0.1% on the skin of the 6 amphibian species before Bd inoculation The lower row represents bacterial genera with relative abundances, and the upper row shows the clustering tree of bacterial genera. The right column indicates the host species.

We used 16S rRNA gene amplicon sequencing to characterize the skin bacterial community of each individual (*Bd* inoculated and control groups, with a total of 318 swab samples) before inoculation (BI) and at the third and sixth weeks postinoculation (3 WPI and 6 WPI) during the periods of peak mortality. After filtration and quality control, we identified 731 operational taxonomic units (OTUs) belonging to 16 phyla in the inoculated groups (Figure S3). Seven phyla, including Proteobacteria, Bacteroidetes, Actinobacteria, Firmicutes, Cyanobacteria, [Thermi], and Planctomycetes, were shared by all the hosts before inoculation, whereas Aquificae was unique to *B. gargarizans*, *Tenericutes* to *C. orientalis*, and WPS-2 to *D. melanostictus*. Six other phyla were shared by a subset of hosts (Figure S3). The proportion of unique OTUs ranged from 0.03% in *S. raddei* to 53.85% in *C. orientalis*. OTUs shared by all the hosts accounted for 10.23% of the total abundance in *C. orientalis* and 34.15% in *D. melanogaster* (Figure S4). After inoculation, the dominant phylum in the bacterial communities shifted for *P. nigrimaculatus* and *S. raddei* at 3 WPI and 6 WPI (Figure S3), and the dominant genera shifted for all the hosts (Figures 1, S5A, and S5B).

**Figure 2:**
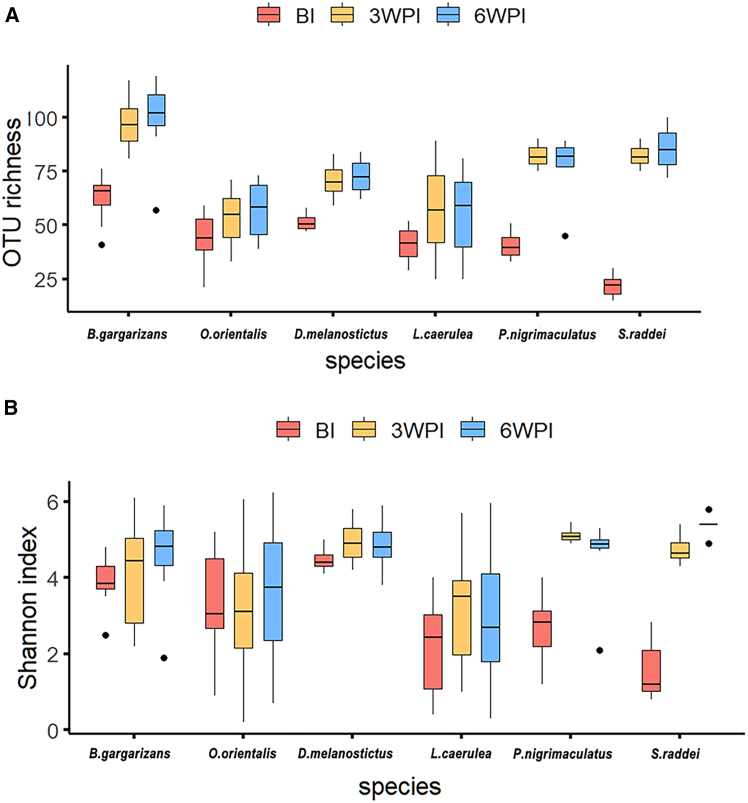
Boxplot of the dynamics of the OTU alpha diversity of the bacterial community compositions on the skin of the 6 amphibian hosts in the Bd-inoculated groups (A and B) (A. OTU richness; B. Shannon index). BI, 3WPI and 6WPI indicate the time points before inoculation and at the third- and sixth-weeks post inoculation, respectively. The black line inside the box indicates the median. The bottom and top borders of the box represent the first and third quartiles, respectively. The vertical lines outside the box represent the upper and lower limits. The outliers are represented as dots.

We investigated the effects of inoculation and hosts on temporal changes in the bacterial alpha diversity of the skin microbiome across inoculated individuals via OTU richness (number of OTUs) and the Shannon index. There were differences in the OTU richness and Shannon index among the hosts before inoculation (Figure 2, Table S4A). *L. caerulea* presented lower OTU richness and Shannon indices than did *B. gargarizans* and *D. melanostictus*, and lower Shannon indices than did *C. orientalis*, but greater OTU richness than did *S. raddei* (Figure 2). A linear mixed-effect model (LMM) suggested that inoculation had a positive effect (standardized coefficient >0) on the OTU richness for all the hosts together but not on the Shannon index (STAR Methods, Table S4B). Each host presented an increase in OTU richness postexposure (Table S4C). Moreover, *L. caerulea* presented lower OTU richness and Shannon index than did *B. gargarizans*, *P. nigrimaculatus,* and *S. raddei* postexposure (Table S4D).

**Figure 3:**
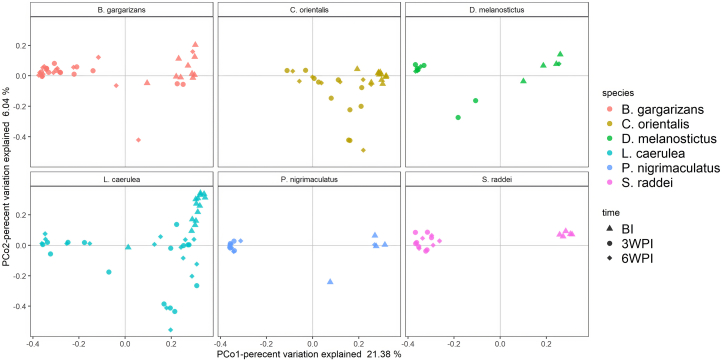
PCoA plots of OTU beta diversity (Bray‒Curtis distance) of bacterial community compositions on the skin of 6 amphibian hosts in the Bd-inoculated groups

We compared the β diversity of bacterial compositions across hosts over time via the Bray‒Curtis distance. PERMANOVA revealed that host species and time-points had significant effects on the Bray‒Curtis distance (*p* = 0.001 for all) (Figure 3; Table S5A). Furthermore, the interaction effect of species and time-points was significant, suggesting that the effects of host species on the Bray‒Curtis distance change with time. Analysis of similarity (ANOSIM) revealed that the Bray‒Curtis distance differed between all pairs except the pairs of *B. gargarizans* and *D. melanostictus* before inoculation (Table S5B). There was also a difference in the Bray‒Curtis distance between *L. caerulea and B. gargarizans* at 3 WPI and 6 WPI postexposure and between some pairs of Asian hosts at 3 WPI (Table S5B). The Bray‒Curtis distance tended to increase (R > 0) for each host at 3 WPI or 6 WPI (Table S5C). The PCoA plots revealed increased dispersion in the bacterial community composition for each host after exposure (Figure 3), compared with that before inoculation. We also found that individuals with similar infection burdens had more similar β diversity of skin microbial communities in three of the 6 inoculated species (Mantel test on the association between the distance matrix of the *Bd* load and Bray‒Curtis distance, *p* ≤ 0.05 for *C. orientalis* and *S. raddei* at 3 WPI and for *L. caerulea* at 6 WPI; Table S6).

### Association between *Bd* infection load and skin bacterial operational taxonomic units

We quantified the effects of alpha diversity and the relative abundance of each OTU (≥0.1%) (log (1 + x) transformed) on the *Bd* load (log (1 + x) transformed) across inoculated individuals for five Asian amphibian hosts and susceptible *L. caerulea* via a generalized linear mixed model (GLMM) (STAR Methods). The model revealed that the OTU richness (log transformed), Shannon index, and relative abundances of 16 OTUs had a negative (standardized coefficient <0), but the relative abundances of 9 OTUs had a positive effect (standardized coefficient >0) on the *Bd* load across inoculated individuals (Table S7). OTUs with negative effects included: an unnamed genus (Microbacteriaceae) in Actinobacteria, Flavobacterium, Sediminibacterium, and an unnamed genus (Weeksellaceae) in Bacteroidetes, Bacillus, Clostridium, and an unnamed genus (Aerococcaceae) in Firmicutes, Brevundimonas, Bradyrhizobium, Rhodoplanes, Sphingopyxis, Ralstonia, Escherichia, Acinetobacter, Enhydrobacter, and an unnamed genus (Caulobacteraceae) in Proteobacteria. While the OTU richness and Shannon index explained 4.45% and 6.45% of the deviations in the *Bd* load, respectively, an OTU accounted for 2.62% (Sphingopyxis) to 9.74% (Ralstonia) of the deviation (Table S7). These OTUs had low relative abundances, usually below the middle of the relative abundance over different time-points (Tables S8A–S8C). The OTUs with positive effects on the *Bd* load were Actinobacteria, Bacteroidetes, and Proteobacteria (Table S7).

We identified representative OTUs with negative effects that separate Asian hosts from susceptible *L. caerulea* via the nonparametric linear discriminant analysis effect size (LEfSe) method.^75^ This method is a classification technique that can determine which OTUs are most likely to explain the differences in disease susceptibility between host groups. LEfSe revealed that among the 16 OTUs, Achromobacter before inoculation (Figure S6A), Bradyrhizobium and Sediminibacterium at 3 WPI (Figure S6B), and Rhodoplanes and Sphingopyxis at 6 WPI (Figure S6C), were consistently more abundant (or enriched) in Asian amphibians than *L. caerulea* (LDA scores >2). Figure S7 shows the visualized distribution of the relative abundances of these enriched OTUs across individuals in the inoculated groups over time. Achromobacter and Sphingopyxis were common in Asian hosts but were rare in *L. caerulea* (Figures S7A and S7E), whereas other OTUs generally presented relatively high values of relative abundance compared with those of susceptible frogs (Figures S7B–S7D).

Because alpha diversity included the enriched OTUs with a negative effect on the *Bd* load, we reran GLMMs to determine the unique negative effect of alpha diversity on the *Bd* load via datasets excluding the 5 consistently enriched OTUs in experimental populations of Asian amphibians (OTU richness1 and Shannon index1, Table S7) or excluding the 4 consistently enriched OTUs in both experimental and wild populations of Asian amphibians (OUT richness2 and Shannon index2, Table S7) compared with *L. caerulea* (see later in discussion LEfSe). The negative effects of OTU richness and Shannon index on the *Bd* load still held for both datasets.

### Comparisons of the bacterial microbiome between wild populations of Asian hosts and captive population of *L. caerulea*

We swabbed the skins of 71 postmetamorphic individuals from wild populations of five Asian amphibian species in 2019, including 8 frogs of *B. gargarizans*, *11 D. melanostictus*, *20 P. nigromaculatus*, 19 *S. raddei*, and 13 individuals of *C. orientalis* (STAR Methods, Table S1). Both nested PCR and qPCR assays revealed that all individuals (*n =* 71), populations (*n = 5*), and species (*n = 5*) were negative for *Bd* infection. Figure S8 presents data on the OTU richness and Shannon index for Asian wild populations compared with susceptible *L. caerulea* before inoculation. *L. caerulea* before inoculation presented a lower Shannon index than did the wild population of each Asian host, and lower OTU richness than did the wild populations of *P. nigromaculatus, B. gargarizans,* and *S. raddei* (Table S9). Figure S9 provides data on the Bray‒Curtis distance of the skin bacterial microbiome. *L. caerulea* before inoculation presented a smaller Bray‒Curtis distance than did the wild population for each Asian host (*p* = 0.011 for all) (Table S10).

LEfSe indicated that, similar to those in the experiments, OTUs with negative effects on *Bd* load, such as Achromobacter, Bradyrhizobium, Sediminibacterium and Rhodoplanes across the five wild populations were consistently more abundant than *L. caerulea* before inoculation (LDA>2) (Figure S10), as were other OTUs, such as Enhydrobacter, Ralstonia, Micrococcus, and Escherichia. In contrast, Acinetobacter, Brevundimonas and an unnamed genus, Weeksellaceae, had higher abundances in *L. caerulea* than in all Asian wild populations. Figure S11 shows the relative abundances of these OTUs that were enriched on skin across Asian wild populations compared with *L. caerulea* before inoculation.

## Discussion

Asian amphibians experimentally showed no clinical signs of disease or death, and weaker *Bd* loads, compared with susceptible *L. caerulea*. Furthermore, the structures of the skin bacterial microbiome differed between *L. caerulea* and Asian hosts before inoculation in the experiments or in the wild. Alpha diversity and the relative abundances of 16 OTUs had a negative effect on the *Bd* load across inoculated animals. Among these OTUs, four representative genera (Bradyrhizobium, Achromobacter, Sediminibacterium and Rhodoplanes) were consistently enriched in both inoculated and wild populations of Asian hosts compared with *L. caerulea*. These results suggest that the skin bacterial microbiome of Asian hosts is likely linked to host resistance to *Bd* infection.

The susceptibility of *L. caerulea* to the *Bd*GPL lineage in this study parallels the findings of other studies,^43^ but the mortality within the 100 days-the period post inoculation (6/15 = 40%) was lower than that in Korea (13/15 = 86.7%) (chi-square test, *p* = 0.009), despite doubling the number of *Bd* zoospores used in this study. The differences in mortality between the two studies may be due to differences in the *Bd*GPL strains or populations of *L. caerulea* used in the experiments. Fu and Waldman revealed that three Korean native frogs including *B. gargarizans*, were resistant to the *Bd*GPL and *Bd*Asia-1 lineages.^43^ Our study provides evidence for the resistance of four additional Asian hosts to lineage *Bd*GPL infection.

The negative effect of alpha diversity on the *Bd* load in this study conforms to studies on amphibians in Puerto Rico and France.^24^^,^^76^ High bacterial diversity may promote competition for resources with *Bd* or the production of high concentrations of antifungal metabolites,^24^^,^^76^ which have adverse effects on the *Bd* load. However, the associations between *Bd* infection intensity or prevalence and skin bacterial diversity may not be universal, and host-specific or environmental factors may affect these associations.^60^^,^^69^^,^^77^ For example, a weak positive correlation between *Bd* infection and skin bacterial richness was observed in treefrog species in Brazil’s Atlantic Forest.^60^^,^^78^ In the high-altitude forests of southern China, a positive correlation was identified for a toad species, whereas the opposite trend was observed for a treefrog species.^35^ The positive correlations might be due to a causal link between high *Bd* prevalence and high environmental microbial diversity^60^^,^^78^ or effects of host physiology and genetics.^31^ In addition, a high *Bd* load that exceeds a certain threshold could cause dysbiosis (i.e., disrupted the bacterial homeostatic balance with benefits to the host)^76^^,^^79^ and likely result in negative associations between the *Bd* infection load or prevalence and bacterial richness.

The increased β diversity of the skin microbiome postinoculation across hosts represents a dysbiotic state,^24^ a phenomenon that is frequently observed in other systems, such as the microbiome of patients with alcoholism,^80^ liver cirrhosis,^81^ or reef-building corals,^82^ but has been less reported in *Bd* inoculation experiments.^83^ The increased β diversity may be due to two reasons. First, host physiological and immunological changes accompanied by *Bd* invasion likely affect the bacterial diversity and structure of the amphibian skin microbiome.^23^^,^^25^ Second, *Bd* likely competes with skin bacteria for space and/or resources, which could modify microbiome compositions.^69^ These factors could increase OTU richness, influence the relative abundances of bacterial OTUs, and change the dominant phyla and genera of the bacterial microbiome post inoculation. Consistent with a study on experimental and field populations of the endangered frog *Rana sierrae* in California, USA,^9^ we detected positive associations between the distance matrices of *Bd* load and Bray‒Curtis distance in *C. orientalis*, *S. raddei* and *L. caerulea* (Table S6A), suggesting that *Bd* load plays an important role in reshaping the bacterial microbiome structures of these species.

The four representative OTUs were common in the skin bacterial microbiome of amphibians. Isolates of Achromobacter from the skin of the resistant frog *Craugastor fitzingeri* in Panama inhibited *Bd* growth *in vitro*. This genus is common on the skins of free-living treefrogs in Panama and Madagascar,^27^^,^^67^ and on the skins of four native species in India.^34^ Bradyrhizobiaceae (including Bradyrhizobium) generally strongly inhibits *Bd in vitro.*^84^ Chitinophagaceae (Sediminibacterium) is abundant on the skin of wild and captive amphibians in Costa Rica and Panama,^14^^,^^85^ whereas Hyphomicrobiaceae (Rhodoplanes) is prevalent in Panama amphibians. As the inoculated animals in this study were provided sterile water and consistent temperatures during the experiments (see STAR Methods), the four enriched OTUs on Asian hosts were unlikely to be sourced from environmental bacterial reservoirs or were unlikely to be due to environmental filtering.^23^^,^^27^^,^^29^ Asian hosts likely favored the four OTUs due to long-term *Bd* endemicity, which possibly provides protection against *Bd* infection.

Chytridiomycosis threatens numerous rare or endangered amphibians worldwide.^12^ Improving the ability to predict disease susceptibility in free-living amphibians and selecting effective probiotic therapies are crucial for amphibian conservation. Four representative OTUs were stable and predictable in terms of relative abundance across Asian hosts in both the experimental and wild populations and were likely reliable biomarkers for low *Bd* loads. Currently, few defensive bacteria (such as *Janthinobacterium lividum*) have been used for probiotic treatment, and their effectiveness varies from preventing the disease to lacking an effect, depending on the host species.^14^^,^^22^

### Limitations of the study

This study has several limitations. As multiple endemic *Bd* lineages exist in Asia,^23^^,^^37^^,^^39^ there is a need to test the virulence of these lineages in Asian amphibians and understand the dynamics of the skin microbiome inoculated with the lineages in controlled experiments. As all animals sampled from the wild were *Bd* negative in this study, the associations among *Bd* infection, the structure of the skin microbiome, and ecological factors in the wild remain to be examined in the future. This study also lacked functional analysis to confirm the protective role of the four representative OTUs. Understanding the functions of skin microbial communities, and how host immunity and environmental factors modulate skin microbial functions will provide detailed knowledge on the precise role of the skin microbiome in protecting Asian amphibians against *Bd* infection.^69^^,^^86^ Identifying these OTUs and elucidating their resistance mechanisms will provide not only new resources for developing amphibian probiotics, but also insights into complex interactions among hosts, symbionts and pathogens.

## Resource availability

### Lead contact

Further information and requests for resources and reagents should be directed to and will be fulfilled by the lead contact, Yiming Li (liym@ioz.ac.cn).

### Materials availability

All the requests for the frog samples and data should be directed to the lead contact and will be made available on request after the completion of a Materials Transfer Agreement.

### Data and code availability

• All microbiota sequencing data generated in this study have been deposited in Mendeley Data (https://doi.org/10.17632/w6rxxvkkzy.1).
• All codes have been deposited in Mendeley Data (https://doi.org/10.17632/w6rxxvkkzy.1).
• Any additional information required to reanalyze the data reported in this article is available from the lead contact upon request. Correspondence and requests for materials should be addressed to YL (liym@ioz.ac.cn).

## Acknowledgments

This work is supported by grants from the 10.13039/501100001809National Science Foundation of China (32030070), the 10.13039/501100003787Hebei Natural Science Foundation (C2022201042), the Second Tibetan Plateau Scientific Expedition and Research (STEP) Program (2019QZKK0501), the High-Level Talents Research Start-Up Project of Hebei University (050001-521100222045), and the China’s Biodiversity Observation Network (Sino-BON).

## Author contributions

YL conceived and designed the study; XY, JZ, SY, XH, SW, CX, TD, XL, and YL collected the data. XY, JZ, YL, XAH, TS, and MQ analyzed the data; YL, JZ, XY, XAH, TG, and MF wrote the article.

## Declaration of interests

The authors have no financial conflicts of interest.

## STAR★Methods

### Key resources table

**Table:** 

REAGENT or RESOURCE	SOURCE	IDENTIFIER
**Biological samples**		

Frog samples	Sampled in this study	See Figure S1; Table S1

**Chemicals, peptides, and recombinant proteins**		

DNA polymerase	TransGen	TransStart® FastPfu DNA Polymerase
*Bd* strains	Inoculated in this study	YSF01, see Figure S12 for details
Data	Used in this study	All sequencing data generated in this study have been deposited in Mendeley Data (https://doi.org/10.17632/w6rxxvkkzy.1)
Code	Used in this study	All codes have been deposited in Mendeley Data (https://doi.org/10.17632/w6rxxvkkzy.1).

**Software and algorithms**		

R Version4.2.2	R Core Team	https://www.r-project.org/index.html
QIIME 2	Hall and Beiko	https://qiime2.org
FSTAT v2.9.4	Jérôme Goudet	https://www2.unil.ch/popgen/softwares/fstat.htm
Arlequin 3.11	Excoffier, L. G. Laval, and S. Schneider	http://cmpg.unibe.ch/software/arlequin3/
SPSS 21.0	IBM Corp	https://www.ibm.com/support/pages/node/609527
MEGA7	Molecular Biology and Evolution	https://www.megasoftware.net/
PartitionFinder v2.1.1	Molecular Evolution and Phylogenetics	https://www.robertlanfear.com/partitionfinder/

### Experimental model and study participant details

#### Sampling and maintenance

We obtained postmetamorphic individuals of *C. orientalis* and *L. caerulea* from pet markets in Beijing (Table S1; Figure S1), and collected postmetamorphic individuals of the other four Asian species from the wild in Beijing, Hengshui of Hebei Province in the temperature plain region, and Lishui College of Zhejiang Province in the subtropical plain region, mainland China, from 2015 to 2016. We collected each individual by hand with new pair of nonpowdered latex gloves that were discarded after each animal was examined. We rinsed each individual with 50 mL of sterile water, and then swabbed it via two swabs (one for the *Bd* test and one for the microbiome test) with a standard number of strokes (five times per foot, each side of the belly, and mouth, for a total of 40 strokes) upon collection or purchase.^33^ The frogs were placed in separate plastic bags with holes (for air flow) and returned to the laboratory. Each swab in the laboratory was examined via a nested PCR assay following the protocol of Goka and colleagues^19^^,^^36^^,^^37^^,^^45^ (see below). All animals were *Bd* negative according to the assay. Each animal was housed individually in a 20 × 12 × 15 cm closed plastic box (sterilized before use) in a laboratory maintained at 21(±2)°C with a 12 L: 12 D photoperiod for at least two weeks before *Bd* inoculation to standardize the environmental conditions. Each box contained 200 mL of sterile water and a stone as land for rest. Each animal was fed 3 crickets weekly (commercial crickets (*Gryllus bimaculatus*), approximately 15 mm in length, vitamin-dusted with calcium powder^87^), and the water in each box was changed twice a week. Body mass and snout-vent length (SVL) were measured before and after the experiments.

We also rinsed and swabbed each postmetamorphic individual from the wild populations of the four Asian amphibians at the same sites where the experimental animals were collected, and from *C. orientalis* in Qianping Anhui Province (subtropical mountain region), in April–May 2019 (Table S1). All experimental and field procedures were approved by the institutional Animal Care and Use Committee of The Institute of Zoology, The Chinese Academy of Sciences.

### Method details

#### *Bd* inoculation experiments

We inoculated amphibians with the strain YSF01 of *Bd*GPL lineage (Figure S12). The strain was isolated from the skin of infected *Xenopus laevis* and cultured in TGhL agar (16 g tryptone, 4 g gelatin hydrolysate, 2 g lactose, 12 g agar, and 1 L distilled water).^88^ We harvested *Bd* zoospores by flooding each plate with 1–2 mL of sterile water and counted the moving zoospores under a microscope via a hemocytometer.

We simultaneously performed a replicated, randomized experiment with six groups (each species as a group) exposed to *Bd* zoospores (treatment) and six groups that were not infected with *Bd* (control) (Table S1). The animals for each host were randomly assigned to the treatment or control group. We inoculated each animal for the treatment groups in a small container (10 × 6 × 5 cm, containing 100 mL of sterile water) supplemented with a solution of approximately 1x10^6^ *Bd* zoospores for 24 h.^89^ Animals in the control groups were treated in the same way but without any microorganisms in sterile water. We then placed the animals back into their original boxes. The experimental animals were monitored every 4 h for clinical signs of chytridiomycosis, such as lethargy, cutaneous erythema, inappetence and skin sloughing.^44^ We swabbed each animal with two swabs using new disposable gloves each time before and post inoculation weekly. An animal was not swabbed if it was *Bd* negative for four-consecutive weeks. The experiment for a host species ended at the week when most inoculated individuals (>80%) were *Bd* negative or 40% of inoculated individuals died.

As deaths occurred only in *L. caerulea*, we compared survival rates between the experimental and control groups for the host via the nonparametric Kaplan‒Meier method with a Cox proportional hazards model.^90^ We performed the procedure in the survival and survminer packages in R.

#### *Bd* infection and *Bd* load

We tested *Bd* infection in samples from the wild via nested PCR,^19^^,^^36^^,^^37^^,^^45^ and quantified the *Bd* load in the samples via qPCR,^37^^,^^91^ as described in previous studies. We extracted *Bd* genomic DNA from swabs following the procedure of Zhang et al.^19^ For nested PCR, first-round amplification was performed with the primers ITS1f and ITS4, which amplify the 5.8S rRNA gene along with the flanking internal transcribed spacer (ITS). We then amplified the first-round PCR products via the primers Bd1a and Bd2a.^36^ The optimized protocol for amplification followed the procedure of Zhang et al.^5^ For each amplification, we used sterilized distilled water as a negative control, and DNA containing 0.1 *Bd* zoospore standards as a positive control. Each swab was tested in triplicate and recorded as *Bd* positive if two replicates showed the presence of *Bd*.

For the qPCR, we created standard curves using 100, 10, 1 and 0.1 *Bd* zoospore standards and ran each sample in triplicate. We estimated the *Bd* infection intensity (*Bd* load) as the mean genomic equivalent (GE) score per sample in triplicate. We considered the sample positive if the GE score was >0. The infection intensity was corrected by accounting for the proportion of the swab extract used in the qPCR.^37^^,^^74^

#### 16S rRNA amplicon sequencing and bioinformatics processing

Bacterial genomic DNA was extracted from each swab using the E.Z.N.A. DNA kit (catalog no. D3350-02; Omega Biotek, Norcross, GA, USA) following the manufacturer’s protocol, with slight modifications in the pretreatment of the samples.^33^ We pretreated samples by immersing swabs with 1 mL of 1x TE buffer, vortexing them on a VORTEX-GENIE2 and discarding the swabs. We centrifuged the buffer at 10000xg for 3 min and discarded the supernatant. Then, 100 μL of 1x TE buffer was added to the buffer, which was vortexed to completely resuspend the pellet. The V4 region of the 16S rRNA gene in the DNA extract was amplified via the primers 515F (5′-GTGCCAGCMGCCGCGGTAA-3′) and 806R (5′-GGACTACHVGGGTWTCTAAT-3′) (barcode-515F and barcode-806R).^92^ PCRs were run by Pfu DNA polymerase (TransGen, Beijing, China), including adenaturing step at 95°C for 2 min; 35 cycles of 95°C for 20 s, 51°C for 20 s, and 72°C for 30 s; and the final extension of 5 min at 72°C. The amplification products were sequenced on an Illumina HiSeq platform (Novogene Bioinformatics Technology Company, Beijing, China) via a 250 bp paired-end strategy.^93^

The sequences for all the experimental and field samples were processed via the open-source Quantitative Insights Into Microbial Ecology 2 (QIIME 2 (version 2023.09)) pipeline^94^ (https://qiime2.org). We visualized sequence quality and denoised the sequences via the default parameters. Sequences shorter than 250 bp were discarded.^27^ We classified the sequences via the naive Bayes classifier artifact and the sci-kit-learn Python library. The classifier artifact is trained on Greengenes2,^95^ trimmed to the V4 hypervariable region via the primers 515f/806r, and clustered at 99% sequence identity. We deleted the sequences derived from chloroplasts, mitochondria, archaea and eukaryotes from the taxonomic units, and then removed the OTUs covering <0.005% of the total reads.^96^ The number of reads per sample was uneven, ranging from 21,420 to 154,799 sequences per sample. We therefore rarefied all the samples to 21,420 reads.^97^

### Quantification and statistical analysis

#### Statistical analysis

We calculated the OTU richness, Shannon index and Bray‒Curtis distance^9^ in QIIME 2. We tested differences in alpha diversity (OTU richness and the Shannon index) among host species before inoculation based on linear models and paired tests with Bonferroni correction. The linear models included the OTU richness or Shannon index as the response variable and species as the independent variable. We investigated the effects of inoculation on alpha diversity postexposure for all hosts together via linear mixed models (LMMs). The LMMs included the OTU richness or Shannon index as a response variable and inoculation (binary variable, samples before inoculation = 0, samples post inoculation (3WPI or 6WPI) = 1) as a predictor variable. To control for phylogenetic-autocorrelations among samples, individual ID (ID) and species were entered into the models as nested random variables (ID/species), and time (BI,3WPI and 6WPI) was included in the models as a random variable. If inoculation was significant for a measure of diversity, we then identified the effects of inoculation on the measure for each host via LMMs with OTU richness or the Shannon index as response variable and inoculation as a predictor variable. ID and time (3WPI or 6WPI) were included in the models as random variables. We examined differences in alpha diversity postexposure among host species via LMMs combined with paired tests. The LMMs included the OTU richness or Shannon index as a response variable and species as a predictor variable. ID and time (3WPI or 6WPI) were entered into the models as random variables.

We examined differences in Bray‒Curtis distance among inoculated groups over time via permutational multivariate analysis of variance (PERMANOVA) with two factors with 999 permutations.^9^ Species and time-points were entered as two factors and the combination of two factors was entered as the interaction. PERMANOVA with one factor was also applied to test differences in Bray‒Curtis distance among wild populations or between wild populations and the *L. caerulea* inoculated group. We performed multiple comparisons of the Bray‒Curtis distance between paired populations via analysis of similarity (ANOSIM).^98^ We conducted a principal coordinate analysis (PCoA) based on the Bray‒Curtis distance to visualize the patterns in β diversity among host species,^23^ over time, or among wild populations. We created a heatmap with the community structure of host species based on OTUs with relative abundances >0.1%.^23^ These analyses were performed via QIIME 2.

We examined the associations between the distance matrices of the *Bd* load and those of the bacterial community composition (β diversity) across individuals inoculated via the Mantel test^9^ in QIIME 2.

We determined the quantitative associations between the *Bd* load and alpha diversity or the relative abundance of each OTU (based on a relative abundance >0.1% at 3 WPI or 6 WPI) via a linear mixed-effects model (GLMM) with a Gaussian error distribution,^99^ with the *Bd* load as the response variable and the Shannon index or the relative abundance of each OTU as a predictor variable. The OTU richness, *Bd* load and relative abundance of each OTU were log (1 + x) transformed to improve their linearity before analysis. *Bd* load and relative abundance of OTUs at each time (point (3 WPI or 6 WPI) were standardized based on *Z* score normalization ((x-mean)/standard deviation). To account for temporal- or source-autocorrelation, time (3WPI or 6WPI) and source (wild or market) were entered as random factors. Individual ID within a species was included as a nested random factor. We calculated the amount of variation (*R*^*2*^_*m*_) explained in the *Bd* load by OTU richness and the Shannon index or an OTU’s relative abundance via model averaging analysis.^100^ We performed LMM and pair tests via ‘lmer’ in the lme4, lmerTest and emmeans packages. We conducted GLMM via ‘glmmTMB’ in the glmmTMB package, and calculated *R*^*2*^_*m*_ via ‘r.squaredGLMM’ in the MuMIn package. These analyses were conducted in R.^101^

We identified the representative bacterial taxa that most likely explained the differences between Asian hosts and susceptible *L. caerulea* from the OTUs with a negative effect on *Bd* load via the linear discriminant analysis effect size (LEfSe) method.^75^ LEfSe identifies representative features (organisms, genes, or taxa) with significant differential abundance between groups via the nonparametric factorial Kruskal–Wallis (KW) sum‒rank test and performs pairwise tests via the Wilcoxon rank‒sum test. The effect size of each differentially abundant feature was estimated via linear discriminant analysis (LDA). LEfSe has been widely used in studies on animal and human microbiomes.^75^^,^^102^ We treated samples of Asian hosts as a group and samples of *L. caerulea* as a susceptible group. We performed such analysis to identify differentially abundant taxa between Asian hosts and *L. caerulea* in experiments at different time-points, and between Asian hosts in the wild and the frog *L. caerulea* in experiments before inoculation on the Galaxy Web platform. The taxa with LDA scores >2 were considered significant.^75^

#### Statistics

Statistical analysis was performed via R software. Statistical significance was defined as *p* < 0.05 (∗), *p* < 0.01 (∗∗), and *p* < 0.001 (∗∗∗) according to two-tailed tests.

## Supplemental information

Document S1. Figures S1–S8 and Tables S1–S3, S5–S7, S9, and S10

Table S4. Effects of inoculation and hosts on temporal changes in bacterial alpha diversity of skin microbiome across inoculated individuals

Table S8. The relative abundance of each OTU at the genus level on amphibian skin for host species in experiments
