# Supplementary material for: Associations between skin bacteria and chytrid fungal infection in Asian amphibians
Source: iScience. 2025 Sep 29;28(11):113661. doi: 10.1016/j.isci.2025.113661 (PMC12549381; doi:10.1016/j.isci.2025.113661)
Supplement: Document S1. Figures S1–S8 and Tables S1–S3, S5–S7, S9, and S10 [file mmc1.pdf]

iScience, Volume *28*

Supplemental information

Associations between skin bacteria and chytrid fungal infection in Asian amphibians

Jiaqi Zhang, Xuejiao Yang, Xavier A. Harrison, Shaofei Yan, Xianglei Hou, Supen Wang, Cunxia Xu, Teng Deng, Tianjian Song, Mingshuo Qin, Xuan Liu, Trenton W.J. Garner, Matthew C. Fisher, and Yiming Li

Supplementary Materials

Fig S1-12

Fig S1. Map of sampling locations (red circles) for five Asian amphibian species.

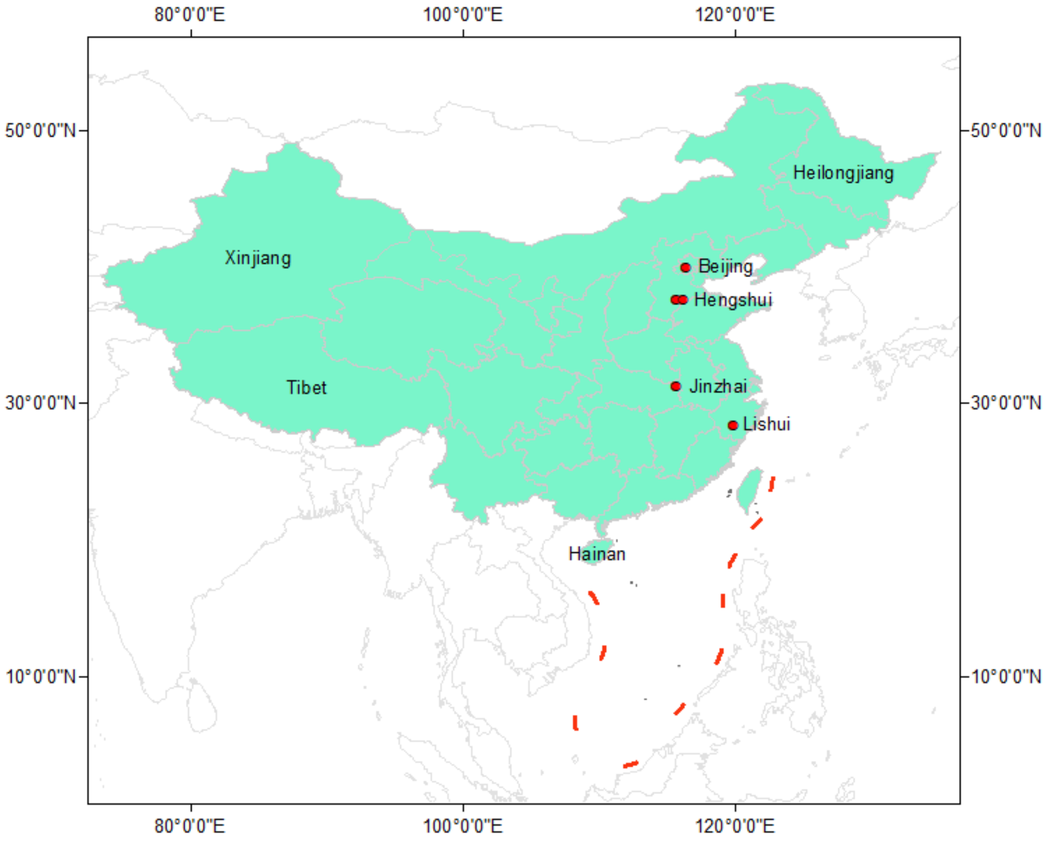

Fig S2. The survival rates and and the dynamics of average *Bd* loads of *L. caerulea*. (A) The survival rates of *L. caerulea* (log-rank test, p=0.003) in *Bd* inoculation experiments and the dynamics of average *Bd* loads per sample for 6 host species across 10 weeks (B). In B, *L. caerulea* included all samples of 15 individuals (sampling on the death between two individual weeks was treated as that of the later week); *L. caerulea* survival included samples of 8 alive individuals; *L. caerulea* dead included samples of 7 dead individuals.

Fig S2A

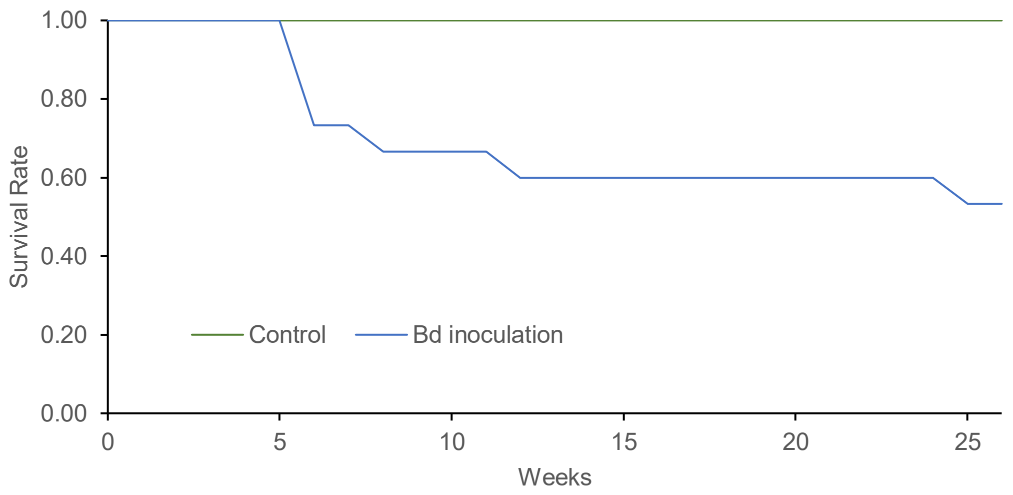

Fig S2B

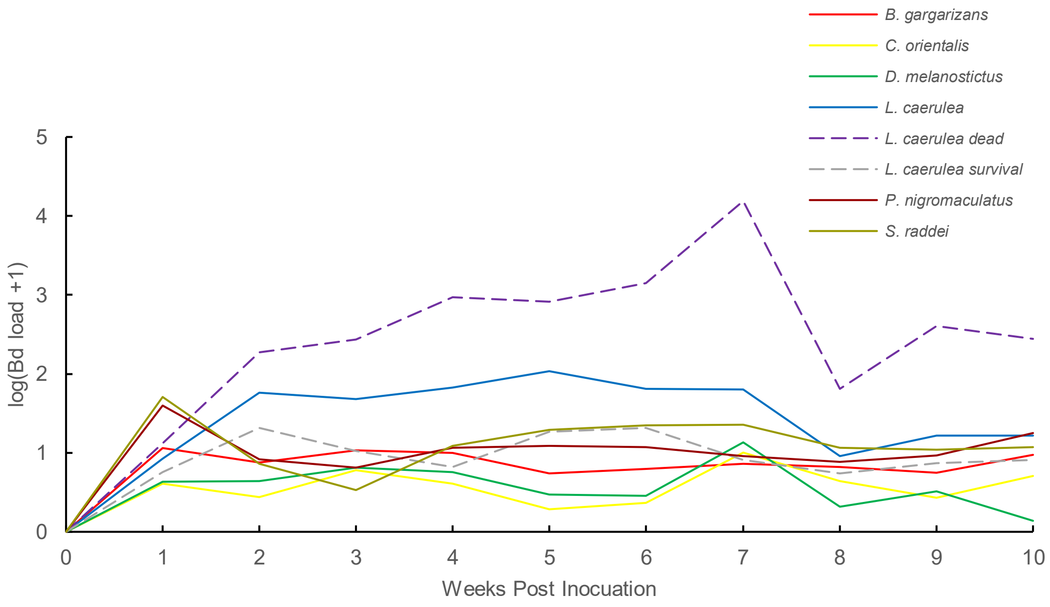

Fig S3. Phylum-level relative abundances of bacterial taxa on skins of 6 amphibian species in inoculated groups before inoculation and post inoculation. BI, 3WPI and 6WPI indicate the time-point before inoculation, at the third and the sixth week post inoculation, respectively.

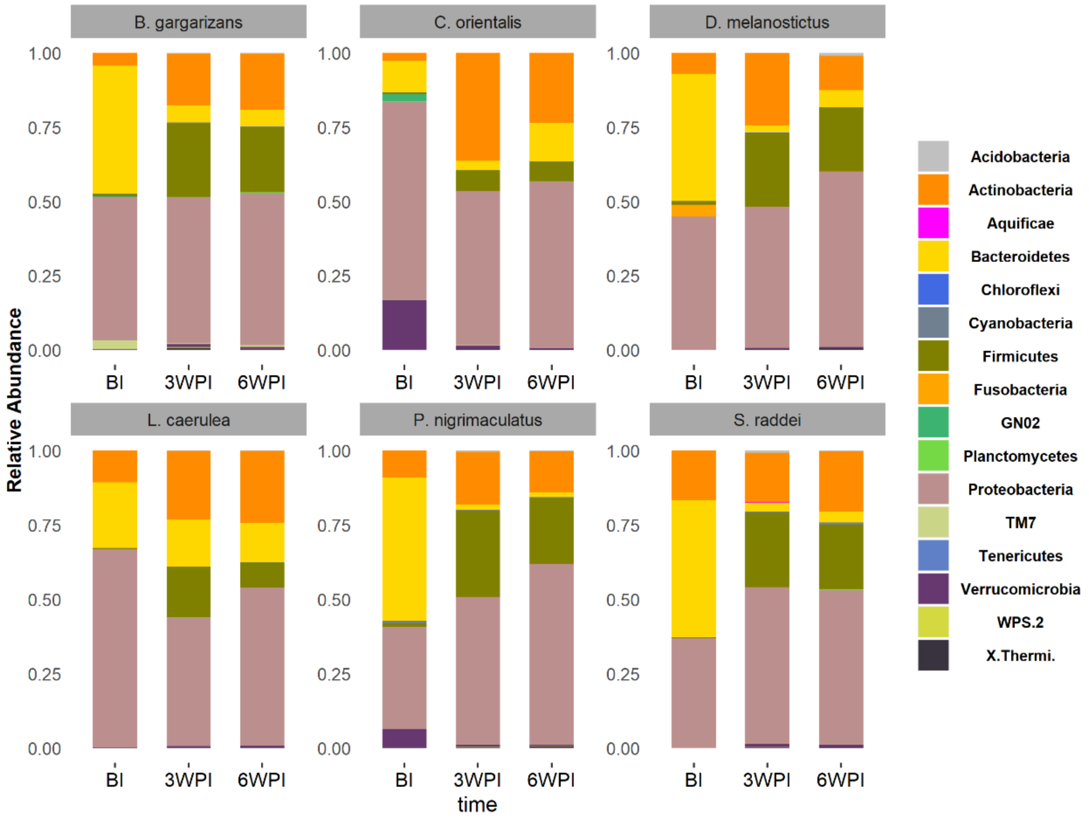

Fig S4. Relative abundances of shared and unique OTUs on skins of 6 amphibian hosts for *Bd* inoculated groups before inoculation.

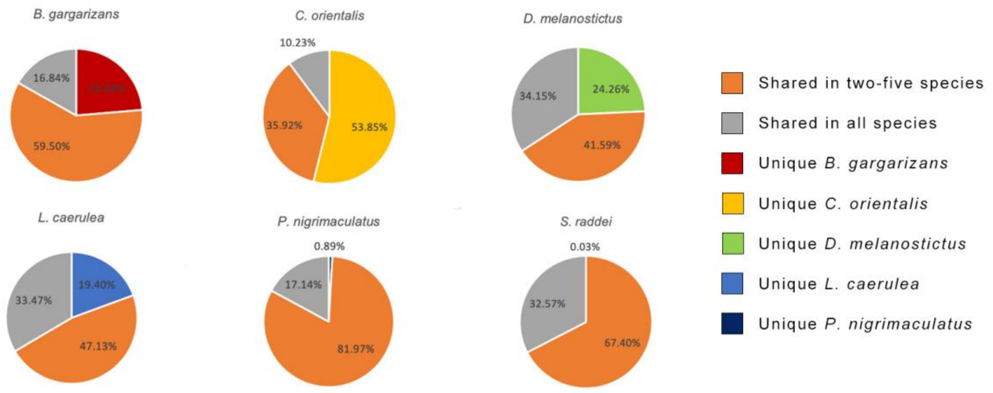

Fig S5. Heatmap of bacterial genera with relative abundances > 0.1% on skins of 6 host species. A, abundances > 0.1% at the third week post inoculation; B, abundances > 0.1% at the sixth week post inoculation. The lower row represents bacterial genera with relative abundances and upper part shows the clustering tree of bacterial genera. Right column indicates the host species.

Fig S5A

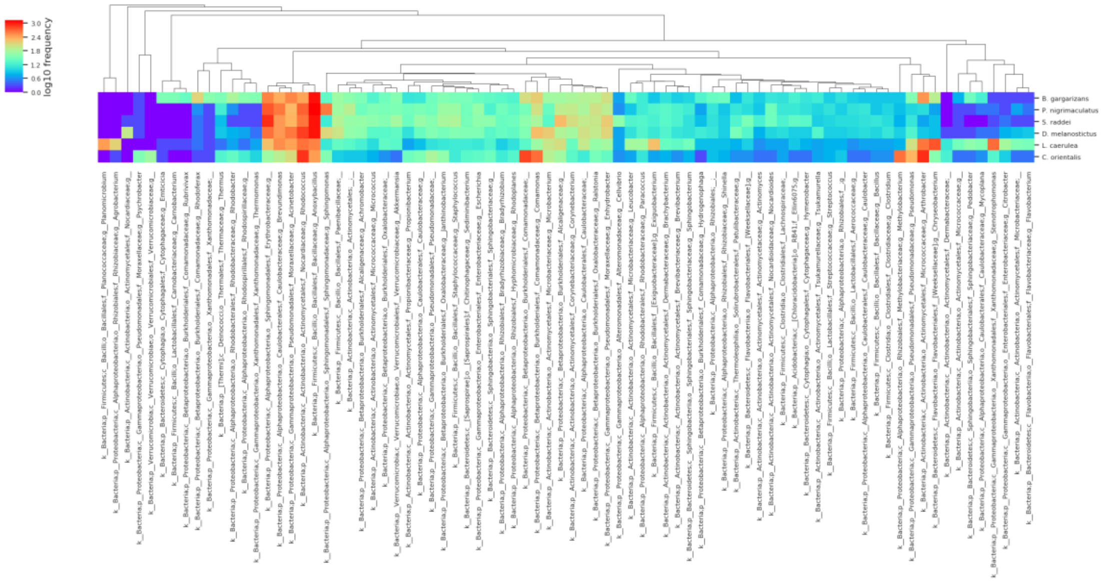

Fig S5B

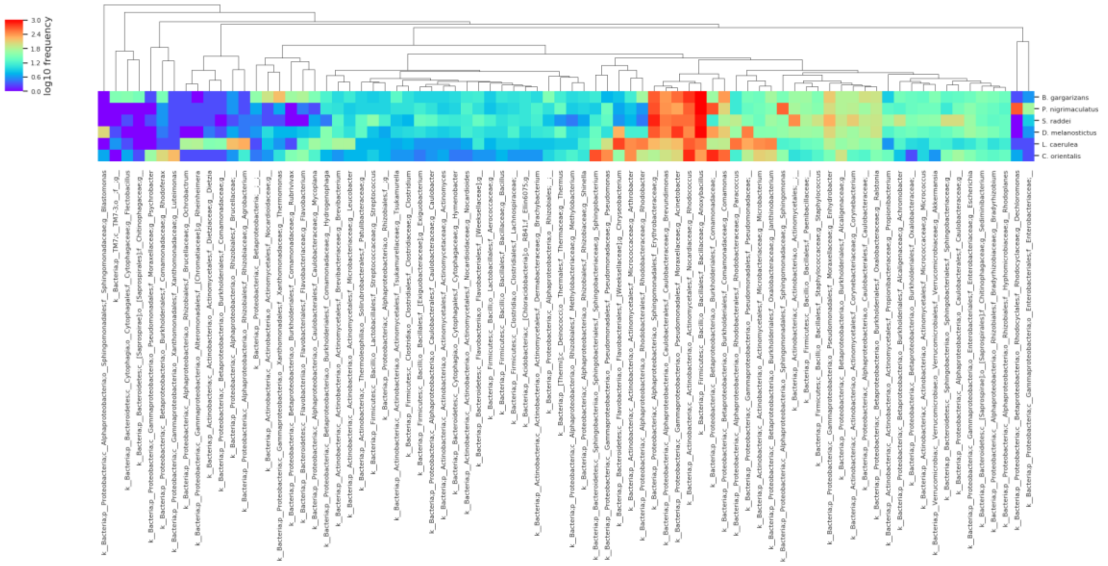

Fig S6. OTUs with negative effects on *Bd* load that differ between five Asian hosts as a group and *L. caerulea* (the susceptible group) in *Bd*-inoculated animals based on LEfSe analysis. A, before *Bd* inoculation; B, at the third week post inoculation. C, at the sixth week post inoculation.

Fig S6A

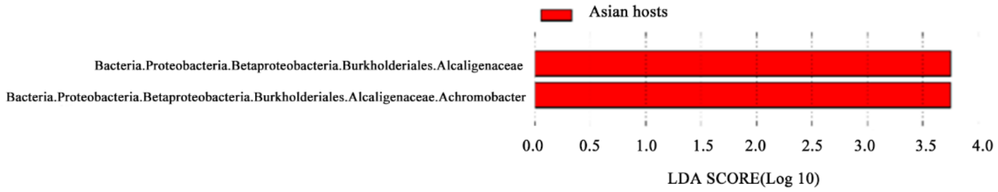

Fig S6B

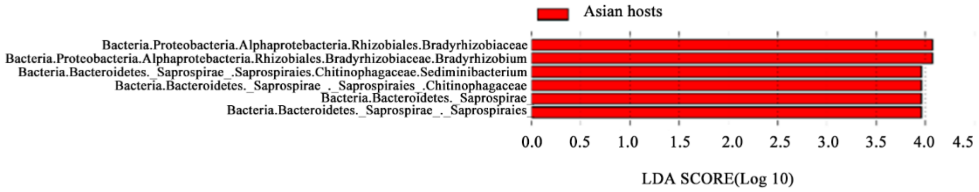

Fig S6C

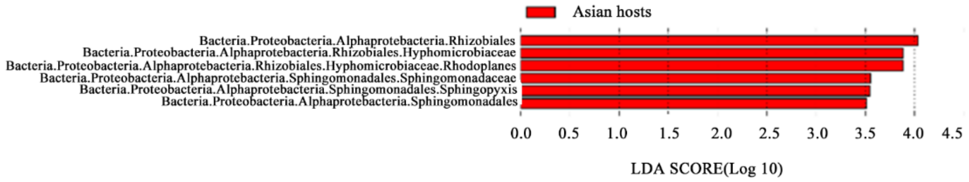

Fig S7. Visualized relative abundances of representative OTUs that were enriched on skins of five Asian hosts compared with *L. caerulea* (the susceptible group) in *Bd*-inoculated animals based on LEfSe analysis. A, Achromobacter before *Bd* inoculation; B, Bradyrhizobium at the third week post inoculation; C, Sediminibacterium at the third week post inoculation; D, Rhodoplanes at the sixth week post inoculation; E, Sphingopyxis at the sixth week post inoculation.

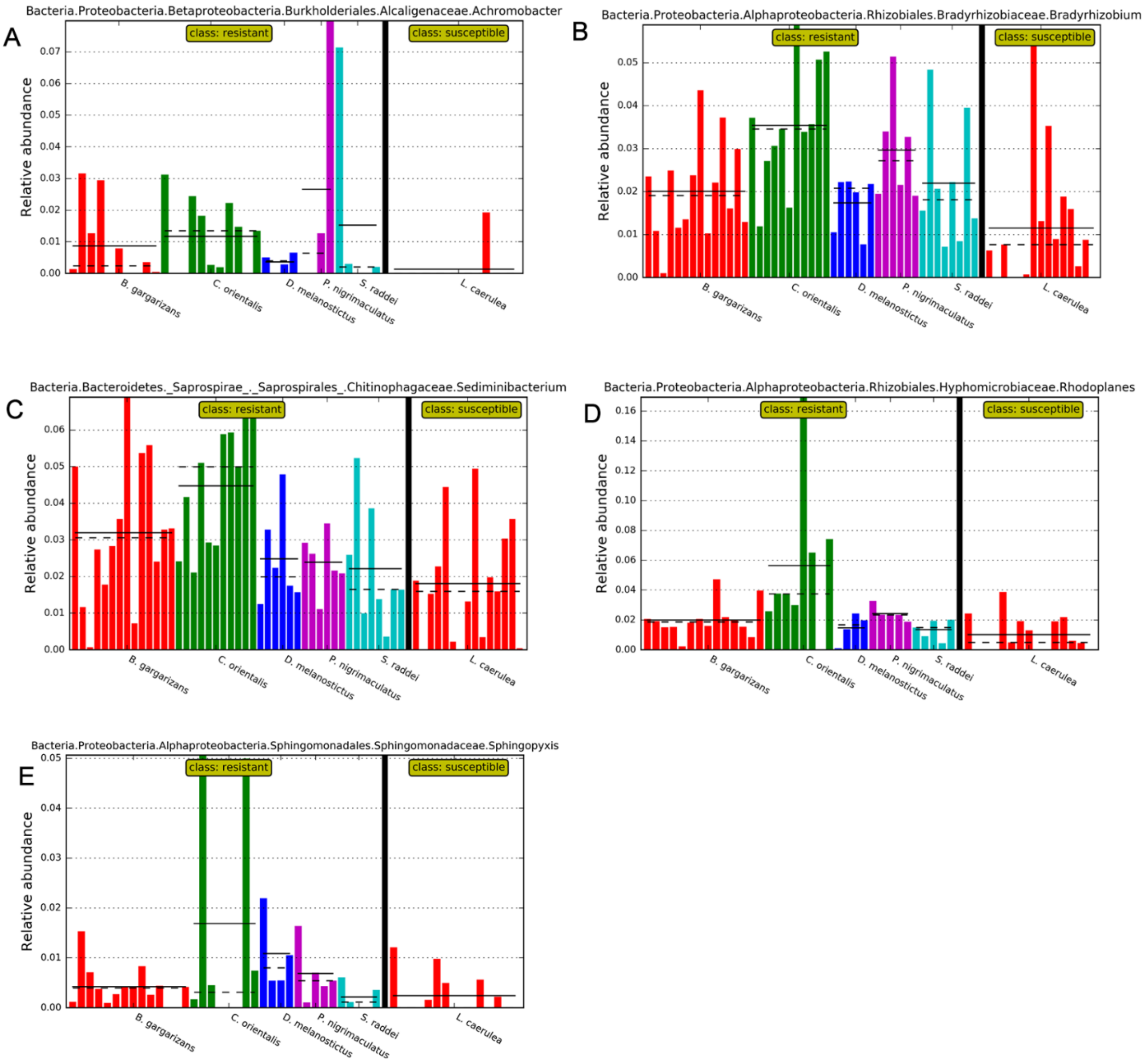

Fig S8. Box plot of OTU richness and Shannon index among wild populations of five Asia hosts and *L. caerulea* before *Bd* inoculation. The black line inside the box indicates the median. The bottom and top borders of the box represent the first and third quartiles. The vertical lines outside the box represent the upper and lower limits. The outliers are represented as dots. A, OTU richness; B, Shannon index.

Fig S8A

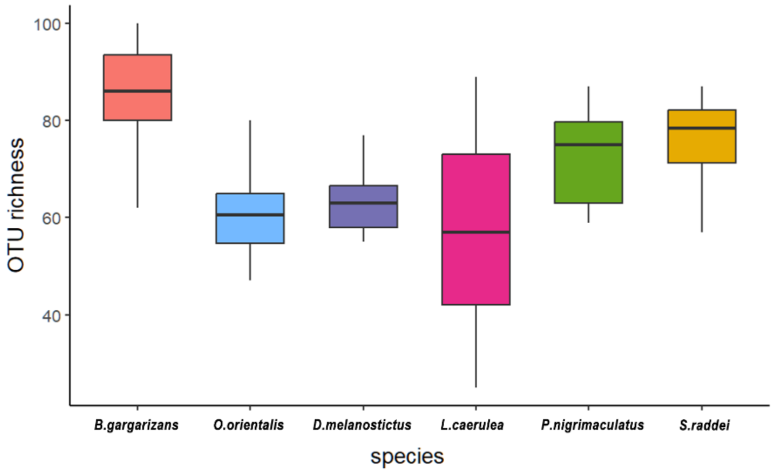

Fig S8B

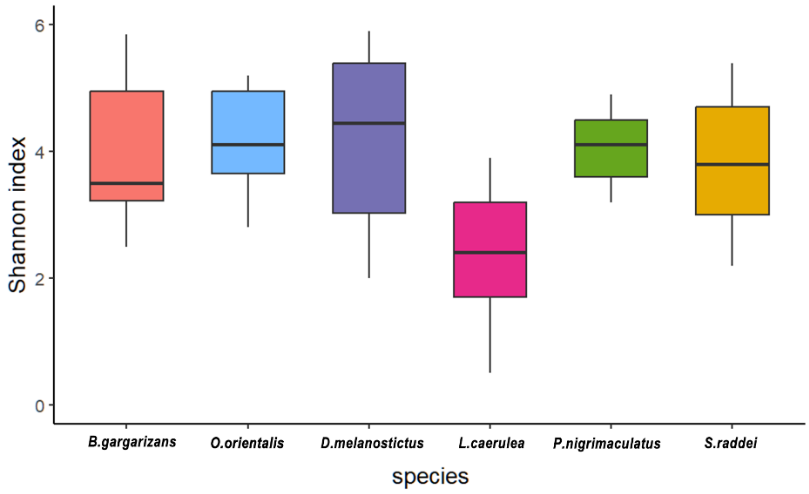

Fig S9. Comparisons on Bray-Curtis distance among wild populations of five Asia hosts and *L. caerulea* before *Bd* inoculation.

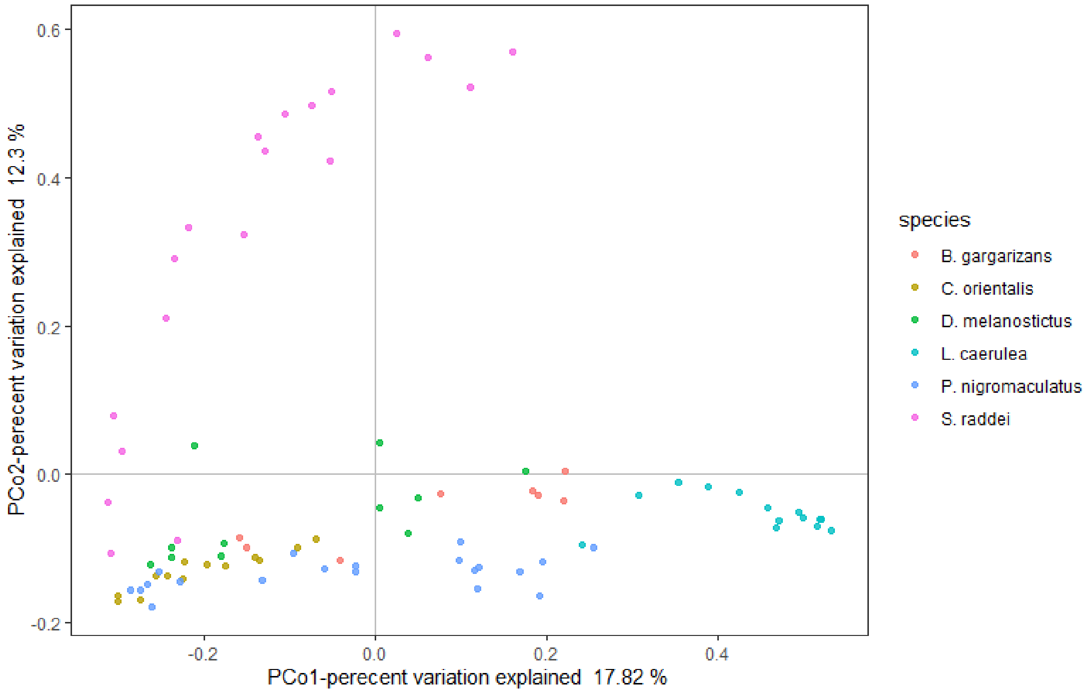

Fig S10. OTUs with negative effect on *Bd* load that differ between wild populations of five Asian hosts as a group and *L. caerulea* (the susceptible group) based on LEfSe analysis.

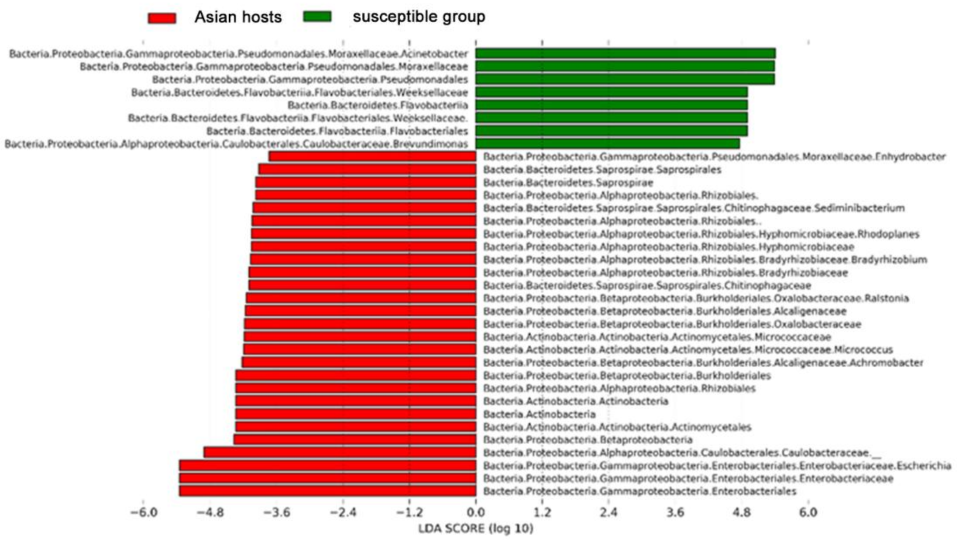

Fig S11. Visualized relative abundances of representative OTUS that were enriched on skins of wild populations of five Asian hosts (as a group) compared with *L. caerulea* (the susceptible group) before *Bd* inoculation.

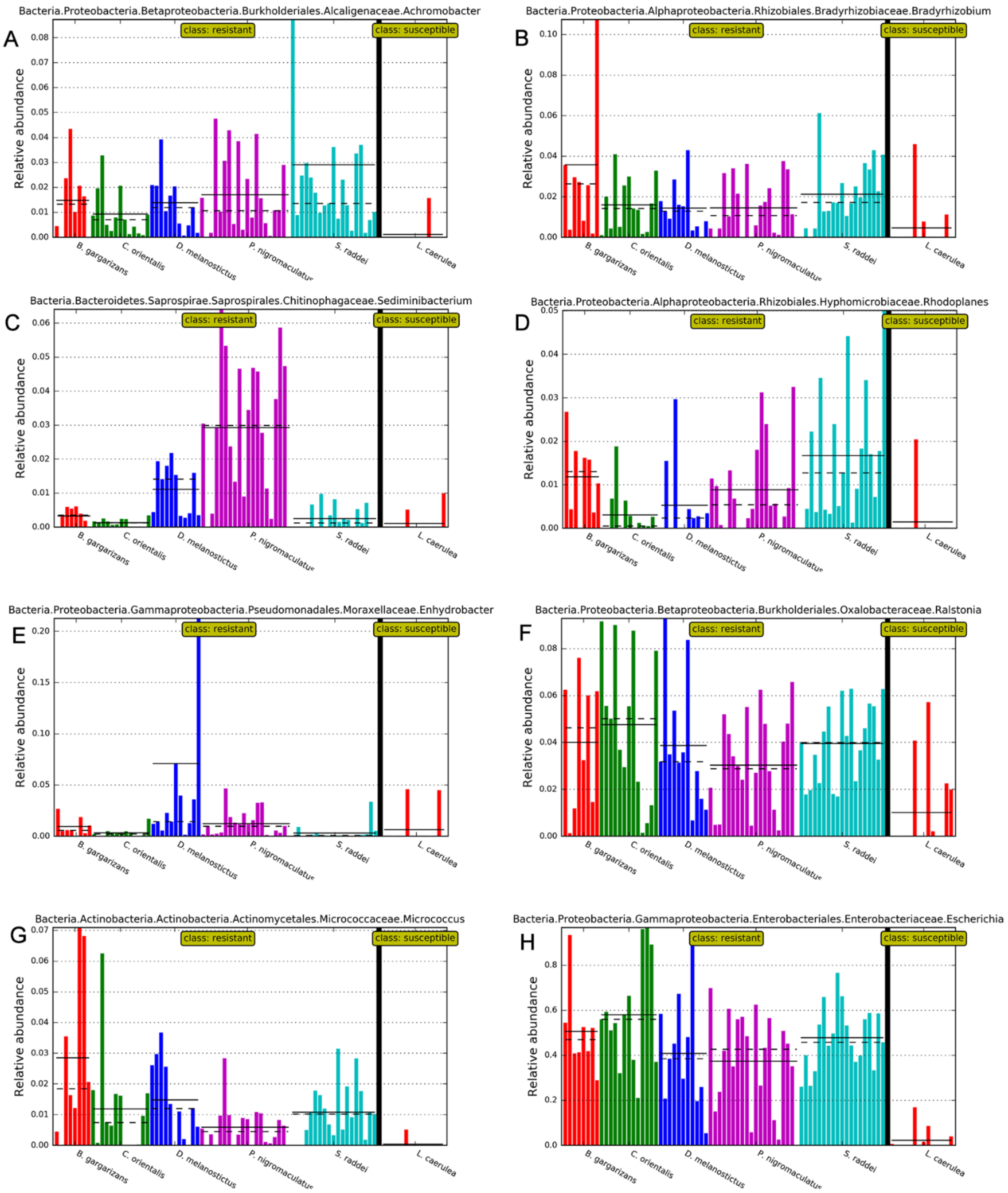

Fig S12. The neighbor-joining distance tree of worldwide *Bd* strains based on multilocus sequence typing of 19 loci. Orange, blue and green represent *Bd*-GPL, *Bd*-Brazil and *Bd*-Korea lineages, respectively. Red indicates the strains isolated in this study.

Two Bd isolates (YSF01 and YSF02) were isolated from the skin of infected *Xenopus laevis*. Multilocus sequence typing (MLST) is based the protocols in previous studies with the 19 loci: APRT13, 6873X2, 8009X2, 8392X2, b7-10c, BDC5, BDC24, BdSC1.2, BdSC1.2.4, BdSC2.02, BdSC3.1, BdSC4.3, BdSC4.16, BdSC5.1, BdSC6.15, BdSC6.2, BdSC8.10, BdSC9.7 and HMG17 (Morehouse et al. 2003; Morgan et al. 2007; James et al. 2009; Schloegel et al. 2012; Bataille et al.2013). Sequences were identified and genotyped after aligned with the library from Bataille et al., 2013. We built the phylogenetic tree of 50 Bd isolates with data on 19 loci (including 48 isolates in Bataili et al 2013 and 2 isolates we obtained) using the Neighbor-Joining method (Saitou and Nei 1987). We performed the analysis in MEGA X (Kumar et al 2018).

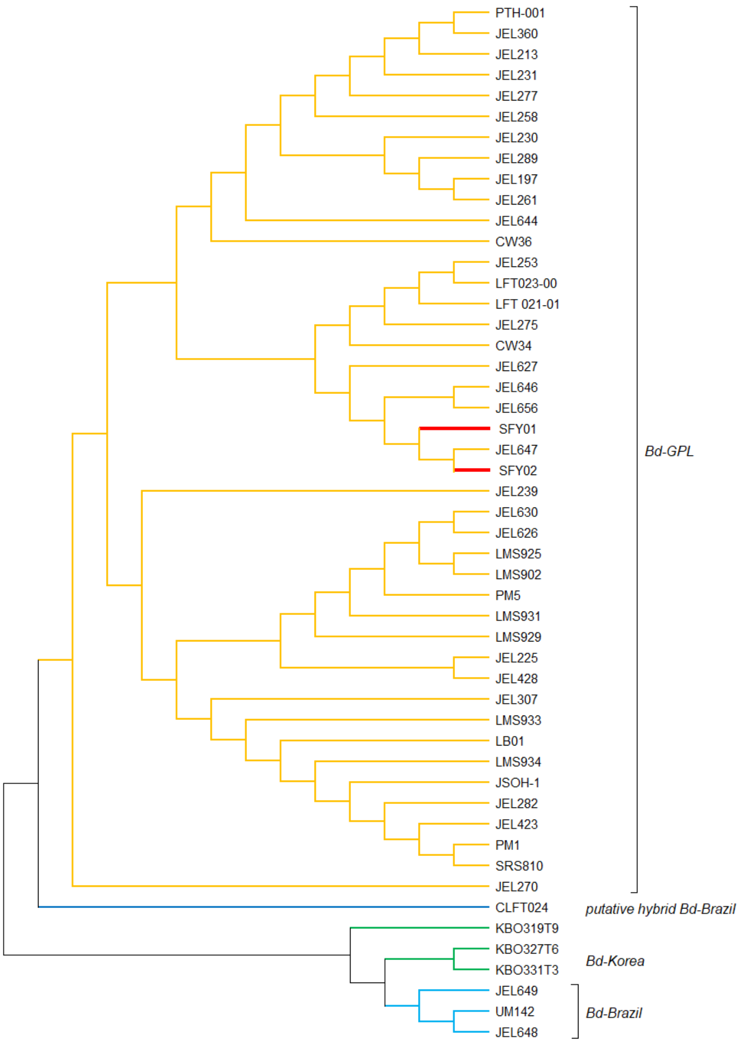

Table S1-10

Table S1. Information on sampling in experiments and wild populations for 6 amphibian species. E=experiments; W=wild population; Sample size for *Bd*-inoculated experiments = number of inoculated individuals/control individuals, and for the wild population = number of individuals swabbed.

Species (family)	Types	Period (days) or date	Sample size (individuals)	Sites (Coordinates: N/E)	Habitats（temperature/humidity)
Bufo gargarizans (Bufonidae)	E	438	15/15	Olympic Park in Beijing (40.015514°/116.378243°)	—
	W	2019/4/18	8	Olympic Park in Beijing (40.015514°/116.378243°)	Ponds in plain (20°C/36%)
Cynops orientalis (Salamandridae)	E	185	12/12	Beijing pet markets	—
	W	2019/4/27	13	Qianping in Anhui Province (31.290498° /115.717036°)	Rice fields in mountains (15℃/98%)
Duttaphrynus melanostictus (Bufonidae)	E	249	8/7	Lishui College in Zhejiang Province (28.465319°/ 119.900567°)	—
	W	2019/4/25	11	Lishui College in Zhejiang Province (28.465319°/ 119.900567°)	Ponds in plain (25℃/93%)
Litoria caerulea (Hylidae)	E	346	15/15	Beijing pet markets	—
Pelophylax nigromaculatus (Ranidae)	E	118	8/8	Hengshui in Hebei Province (37.662760°/115.655905°)	—
	W	2019/5/17	20	Hengshui in Hebei Province (37.662760°/115.655905°)	River in plain (28°C/79%)
Strauchbufo raddei (Bufonidae)	E	187	10/10	Hengshui in Hebei Province (37.659175°/115.638683°)	—
	W	2019/5/22	19	Hengshui in Hebei Province (37.659175°/115.638683°)	Apple orchard in plain (29℃/31%)

Table S2. *Bd* prevalence on 6 host species in experiments. Buga = *B. gargarizans*; Cyor = *C. orientalis*; Dume = *D. melanostictus*; Lica-d = *L. caerulea* dead; Lica-s = *L. caerulea* survival; Peni = *P. nigromaculatus*; Stra= *S. raddei*

Weeks	Buga	Cyor	Dume	Lica-d	Lica-s	Peni	Stra
1 WPI	1	0.917	1	1	1	1	1
2 WPI	1	1	0.875	1	1	1	0.7
3 WPI	1	0.917	1	1	1	1	0.5
4 WPI	1	0.833	1	1	1	1	0.8
5 WPI	0.933	0.583	0.875	1	1	1	1
6 WPI	1	0.833	1	1	1	1	1
7 WPI	1	0.917	0.625	1	1	0.875	1
8 WPI	0.933	0.917	0.5	1	1	0.875	1
9 WPI	1	0.5	0.625	1	1	0.875	1
10 WPI	0.933	0.5	0.625	1	1	1	1

Table S3. Results of ANOVA on average *Bd* loads/sample/week within 6 weeks among host species. *Bd* loads were log (1+x) transformed. Red indicates the significance ≤0.05.

A, tests for differences in *Bd* load among *L. caerulea* and 5 Asian hosts

Test of Homogeneity of Variances

Levene Statistic	df1	df2	Sig.
11.608	5	62	0.000

ANOVA

	Sum of Squares	df	Mean Square	F	Sig.
Between Groups	13.526	5	2.705	10.13	0.000
Within Groups	16.556	62	0.267		
Total	30.082	67			

Multiple Comparisons (Games-Howell test)

species1 (I)	species2 (J)	Mean Difference (I-J)	Std. Error	Sig.	95% Confidence Interval	
					Lower Bound	Upper Bound
B. gargarizans	*C. orientalis*	0.4000	0.1665	0.2010	-0.1219	0.9219
	D. melanostictus	0.2872	0.1701	0.5540	-0.2467	0.8211
	L. caerulea	-0.8791	0.2660	0.0310	-1.7004	-0.0579
	P. nigromaculatus	-0.1758	0.1508	0.8460	-0.6657	0.3141
	S. raddei	-0.2222	0.1656	0.7590	-0.7428	0.2983
C. orientalis	*D. melanostictus*	-0.1128	0.1131	0.9120	-0.4763	0.2507
	L. caerulea	-1.2792	0.2337	0.0000	-2.0258	-0.5326
	P. nigromaculatus	-0.5759	0.0813	0.0000	-0.8428	-0.3089
	S. raddei	-0.6223	0.1062	0.0000	-0.9562	-0.2884
D. melanostictus	*L. caerulea*	-1.1664	0.2363	0.0010	-1.9193	-0.4134
	P. nigromaculatus	-0.4631	0.0884	0.0060	-0.7802	-0.1459
	S. raddei	-0.5095	0.1117	0.0040	-0.8722	-0.1468
L. caerulea	*P. nigromaculatus*	0.7033	0.2229	0.0610	-0.0243	1.4309
	S. raddei	0.6569	0.2331	0.1020	-0.0887	1.4025
P. nigromaculatus	*S. raddei*	-0.0464	0.0794	0.9900	-0.3148	0.2220

* The mean difference is significant at the 0.05 level.

B, tests for differences in *Bd* loads among 5 Asian hosts, *L. caerulea* survival and dead

Test of Homogeneity of Variances

Levene Statistic	df1	df2	Sig.
2.658	6	61	0.023

ANOVA

	Sum of Squares	df	Mean Square	F	Sig.
Between Groups	22.253	6	3.709	28.9	0.000
Within Groups	7.829	61	0.128		
Total	30.082	67			

Multiple Comparisons (Games-Howell)

species1 (I)	species2 (J)	Mean Difference (I-J)	Std. Error	Sig.	95% Confidence Interval	
					Lower Bound	Upper Bound
B. gargarizans	*C. orientalis*	0.4000	0.1665	0.2470	-0.1423	0.9424
	D. melanostictus	0.2872	0.1701	0.6300	-0.2676	0.8421
	L. caerulea dead	-1.6945	0.2195	0.0000	-2.4332	-0.9559
	L. caerulea survival	-0.1657	0.1719	0.9560	-0.7258	0.3945
	P. nigromaculatus	-0.1758	0.1508	0.8960	-0.6856	0.3340
	S. raddei	-0.2222	0.1656	0.8240	-0.7632	0.3188
C. orientalis	*D. melanostictus*	-0.1128	0.1131	0.9470	-0.4910	0.2654
	L. caerulea dead	-2.0946	0.1791	0.0000	-2.7638	-1.4253
	L. caerulea survival	-0.5657	0.1158	0.0030	-0.9543	-0.1771
	P. nigromaculatus	-0.5759	0.0813	0.0000	-0.8538	-0.2979
	S. raddei	-0.6223	0.1062	0.0000	-0.9693	-0.2752
D. melanostictus	*L. caerulea dead*	-1.9818	0.1824	0.0000	-2.6569	-1.3066
	L. caerulea survival	-0.4529	0.1209	0.0280	-0.8657	-0.0400
	P. nigromaculatus	-0.4631	0.0884	0.0070	-0.7943	-0.1318
	S. raddei	-0.5095	0.1117	0.0050	-0.8869	-0.1320
L. caerulea dead	*L. caerulea survival*	1.5289	0.1841	0.0000	0.8515	2.2062
	P. nigromaculatus	1.5187	0.1646	0.0010	0.8467	2.1907
	S. raddei	1.4723	0.1782	0.0000	0.8028	2.1419
L. caerulea survival	*P. nigromaculatus*	-0.0102	0.0919	1.0000	-0.3556	0.3352
	S. raddei	-0.0566	0.1145	0.9990	-0.4444	0.3313
P. nigromaculatus	*S. raddei*	-0.0464	0.0794	0.9960	-0.3261	0.2333

* The mean difference is significant at the 0.05 level.

Table S5. PERMANOVA and ANOSIM for Bray-Curtis distance among host species and over time-points in *Bd* inoculated groups. (A PERMANOVA; B and C ANOSIM)**.** Red indicates the significance ≤0.05. BI, 3WPI and 6WPI indicate the time-point before inoculation, at the third and the sixth week post inoculation, respectively.

A. PERMANOVA for Bray-Curtis distance among host species and over time-points.

Source	Type III Sums of Sqs	Df	Mean Sqs	F. Model	R2	Pr(>F)
Host species	7.138	5	1.428	5.405	0.125	**0.001**
Time-Points	5.846	2	2.923	11.068	0.102	**0.001**
Host species : time-points	6.843	10	0.684	2.591	0.120	**0.001**
Residuals	37.239	141	0.264			
Total	57.066	158				

B. Multiple comparisons (ANOSIM) among host species each time-point. q-value is the corrected p-value by multiple comparisons.

time	Group 1	Group 2	Sample size	R	p-value	q-value
BI	*B. gargarizans*	*C. orientalis*	21	0.8129	0.0010	0.0017
		D. melanostictus	14	0.1887	0.0860	0.0860
		L. caerulea	24	0.6947	0.0010	0.0017
		P. nigrimaculatus	14	0.3608	0.0150	0.0188
		S. raddei	15	0.6924	0.0010	0.0017
	C. orientalis	*D. melanostictus*	15	0.7507	0.0020	0.0030
		L. caerulea	25	0.9583	0.0010	0.0017
		P. nigrimaculatus	15	0.8923	0.0010	0.0017
		S. raddei	16	0.9362	0.0010	0.0017
	D. melanostictus	*L. caerulea*	18	0.5839	0.0010	0.0017
		P. nigrimaculatus	8	0.4740	0.0290	0.0311
		S. raddei	9	0.7625	0.0130	0.0177
	L. caerulea	*P. nigrimaculatus*	18	0.8466	0.0010	0.0017
		S. raddei	19	0.9044	0.0010	0.0017
	P. nigrimaculatus	*S. raddei*	9	0.5438	0.0240	0.0277
3WPI	*B. gargarizans*	*C. orientalis*	25	0.4075	0.0010	0.0075
		D. melanostictus	20	-0.0893	0.7270	0.8950
		L. caerulea	29	0.1134	0.0050	0.0250
		P. nigrimaculatus	20	-0.1530	0.8950	0.8950
		S. raddei	22	-0.0256	0.5670	0.7732
	C. orientalis	*D. melanostictus*	17	0.2762	0.0190	0.0500
		L. caerulea	26	0.0706	0.0920	0.1533
		P. nigrimaculatus	17	0.2950	0.0200	0.0500
		S. raddei	19	0.4795	0.0010	0.0075
	D. melanostictus	*L. caerulea*	21	-0.1567	0.8850	0.8950
		P. nigrimaculatus	12	0.2361	0.0070	0.0263
		S. raddei	14	0.1279	0.0750	0.1406

	L. caerulea	*P. nigrimaculatus*	21	-0.1481	0.8690	0.8950
		S. raddei	23	-0.0315	0.5320	0.7732
	P. nigrimaculatus	*S. raddei*	14	0.1347	0.0660	0.1406
6WPI	*B. gargarizans*	*C. orientalis*	22	0.4104	0.0020	0.0150
		D. melanostictus	18	-0.0341	0.5290	0.6675
		L. caerulea	29	0.1468	0.0020	0.0150
		P. nigrimaculatus	19	-0.0174	0.4870	0.6675
		S. raddei	19	-0.0245	0.4940	0.6675
	C. orientalis	*D. melanostictus*	12	0.0772	0.3080	0.6675
		L. caerulea	23	-0.0272	0.5340	0.6675
		P. nigrimaculatus	13	0.1638	0.0930	0.2790
		S. raddei	13	0.2737	0.0240	0.1200
	D. melanostictus	*L. caerulea*	19	-0.1105	0.6940	0.7436
		P. nigrimaculatus	9	0.0125	0.4150	0.6675
		S. raddei	9	0.0375	0.3150	0.6675
	L. caerulea	*P. nigrimaculatus*	20	-0.0866	0.6650	0.7436
		S. raddei	20	-0.1475	0.8340	0.8340
	P. nigrimaculatus	*S. raddei*	10	0.1500	0.0420	0.1575

C. ANOSIM for Bray-Curtis distance between time-points for each host species.

species	Group 1	Group 2	Sample size	R	p-value	q-value
B. gargarizans	BI	3WPI	24	0.6192	0.0010	0.0015
		6WPI	24	0.6452	0.0010	0.0015
	3WPI	6WPI	28	-0.0135	0.6740	0.6740
C. orientalis	BI	3WPI	22	0.5464	0.0010	0.0015
		6WPI	19	0.5062	0.0010	0.0015
	3WPI	6WPI	19	-0.0162	0.5280	0.5280
D. melanostictus	BI	3WPI	10	0.9365	0.0060	0.0180
		6WPI	8	0.5521	0.0610	0.0915
	3WPI	6WPI	10	-0.0317	0.5610	0.5610
L. caerulea	BI	3WPI	29	0.6609	0.0010	0.0015
		6WPI	29	0.6078	0.0010	0.0015
	3WPI	6WPI	30	0.0304	0.1690	0.1690
P. nigrimaculatus	BI	3WPI	10	0.7381	0.0110	0.0330
		6WPI	9	0.3000	0.0690	0.1035
	3WPI	6WPI	11	0.0693	0.1880	0.1880
S. raddei	BI	3WPI	13	0.9579	0.0020	0.0060
		6WPI	10	0.8400	0.0090	0.0135
	3WPI	6WPI	13	0.0612	0.2760	0.2760

Table S6. Mantel tests for the relationships between Bray-Curtis distance matrices of bacterial communities and distance matrices of *Bd* load across individuals within a population. 3WPI and 6WPI indicate the third and sixth week post inoculation, respectively. Red indicates the significance≤0.05.

species	time	Sample size	Spearman rho	p-value
B. gargarizans	3WPI	14	0.3103	0.088
	6WPI	14	-0.1390	0.458
C. orientalis	3WPI	11	0.4965	0.014
	6WPI	8	0.3120	0.186
D. melanostictus	3WPI	6	-0.0679	0.842
	6WPI	4	-0.4286	0.545
L. caerulea	3WPI	15	0.1178	0.417
	6WPI	15	0.2350	0.020
P. nigrimaculatus	3WPI	6	-0.2821	0.159
	6WPI	5	-0.2918	0.495
S. raddei	3WPI	8	0.2548	0.050
	6WPI	5	0.4182	0.136

Table S7. The relationships between the relative abundance of skin bacterial OTUs and *Bd* load across individuals inoculated for five Asian hosts and susceptible *L. caerulea* based on the GLMMs. The table included the bacterial OTUs with relative abundances > 0.1% on skins of 6 amphibian species (sample size included the samples at the third and the sixth week post inoculation. Red indicates the significance ≤ 0.05. R^2_m is the amount of variations in *Bd* load that is explained by a predictor variable. The results with significant probability ($p \leq 0.05$) were compared with the antifungal isolates databases of Woodhams et al., (2015) (http://www.esapubs.org/archive/ecol/E096/059/) and Bridges (2015). †indicating no record by Woodhams', e/i/n/u indicating enhancing, inhibitory, no significance, unknown in Woodhams' respectively. OTU richness1 or Shannon index1 is calculated using the dataset excluding the 5 consistently enriched in experimental populations of Asia amphibians compared with the experimental population of *L. caerulea*, and OTU richness2 or Shannon index2 using the dataset excluding the 4 consistently enriched genera in both experimental and wild populations of Asian amphibians.

Items	R2m	Estimate	Pr(>|z|)	Reference
OTU richness	0.0445	-0.0077	0.0152	
OTU richness1	0.0420	-0.0077	0.0169	
OTU richness2	0.0433	-0.0076	0.0199	
Shannon index	0.0645	-0.1717	0.0043	
Shannon index1	0.0618	-0.1712	0.0051	
Shannon index2	0.0615	-0.1707	0.0052	
Acidobacteria;[Chloracidobacteria];RB41;Ellin6075;	0.0108	-129.4532	0.239	
Actinobacteria;Actinobacteria;Actinomycetales;__;__	0.0002	2.7081	0.8841	
Actinobacteria;Actinobacteria;Actinomycetales;Actinomycetaceae;Actinomyces	0.0121	-225.9207	0.257	
Actinobacteria;Actinobacteria;Actinomycetales;Brevibacteriaceae;Brevibacterium	0.0568	50.4776	0.0028	i/n
Actinobacteria;Actinobacteria;Actinomycetales;Corynebacteriaceae;Corynebacterium	0.0063	19.9614	0.3717	
Actinobacteria;Actinobacteria;Actinomycetales;Dermabacteraceae;__	0.0025	38.3385	0.5278	
Actinobacteria;Actinobacteria;Actinomycetales;Dermabacteraceae;Brachybacterium	0.0075	-65.1257	0.266	
Actinobacteria;Actinobacteria;Actinomycetales;Microbacteriaceae;__	0.0262	-60.9094	0.0321	i
Actinobacteria;Actinobacteria;Actinomycetales;Dietziaceae;Dietzia	0.0528	69.1214	0.0034	n
Actinobacteria;Actinobacteria;Actinomycetales;Microbacteriaceae;Leucobacter	0.0574	36.0957	0.0092	i
Actinobacteria;Actinobacteria;Actinomycetales;Microbacteriaceae;Microbacterium	0.0044	4.8881	0.413	
Actinobacteria;Actinobacteria;Actinomycetales;Micrococcaceae;	0	1.3533	0.973	
Actinobacteria;Actinobacteria;Actinomycetales;Micrococcaceae;Arthrobacter	0.0233	3.4177	0.0704	
Actinobacteria;Actinobacteria;Actinomycetales;Micrococcaceae;Micrococcus	0.0324	-155.23	0.0575	
Actinobacteria;Actinobacteria;Actinomycetales;Nocardiaceae;	0.0031	15.572	0.5138	
Actinobacteria;Actinobacteria;Actinomycetales;Nocardiaceae;Rhodococcus	0.0002	-0.3188	0.841	
Actinobacteria;Actinobacteria;Actinomycetales;Nocardioidaceae;	0.0001	-1.5064	0.91	
Actinobacteria;Actinobacteria;Actinomycetales;Nocardioidaceae;Nocardioides	0.0044	-72.8724	0.48	
Actinobacteria;Actinobacteria;Actinomycetales;Propionibacteriaceae;Propionibacterium	0.006	-54.594	0.429	
Actinobacteria;Actinobacteria;Actinomycetales;Tsukamurellaceae;Tsukamurella	0.0272	-191.563	0.0597	
Actinobacteria;Thermoleophilia;Solirubrobacterales;Patulibacteraceae;	0.0287	-211.6725	0.0855	
Bacteroidetes;Cytophagia;Cytophagales;Cytophagaceae;Emticicia	0.0063	-32.466	0.31	

Bacteroidetes;Cytophagia;Cytophagales;Cytophagaceae;Flectobacillus	0.0028	-17.9486	0.528	
Bacteroidetes;Cytophagia;Cytophagales;Cytophagaceae;Hymenobacter	0.0007	-33.7625	0.753	
Bacteroidetes;Flavobacteriia;Flavobacteriales;Flavobacteriaceae;Flavobacterium	0.0282	-26.6817	0.0334	e/i/n/u
Bacteroidetes;Flavobacteriia;Flavobacteriales;[Weeksellaceae];	0.0605	-234.7687	0.003	e/i/n/u
Bacteroidetes;Flavobacteriia;Flavobacteriales;[Weeksellaceae];Chryseobacterium	0.1355	8.634	0	e/i/n/u
Bacteroidetes;Sphingobacteriia;Sphingobacteriales;Sphingobacteriaceae;	0.0002	-4.45	0.866	
Bacteroidetes;Sphingobacteriia;Sphingobacteriales;Sphingobacteriaceae;Pedobacter	0.0015	-10.5053	0.621	
Bacteroidetes;Sphingobacteriia;Sphingobacteriales;Sphingobacteriaceae;Sphingobacterium	0	0.1454	0.968	
Bacteroidetes;[Saprospirae];[Saprospirales];Chitinophagaceae;	0.0035	-24.9454	0.476	
Bacteroidetes;[Saprospirae];[Saprospirales];Chitinophagaceae;Sediminibacterium	0.0557	-136.7768	0.0057	†
Firmicutes;Bacilli;Bacillales;Bacillaceae;Anoxybacillus	0.0215	-3.4035	0.125	
Firmicutes;Bacilli;Bacillales;Bacillaceae;Bacillus	0.0611	-440.2504	0.0024	i/n
Firmicutes;Bacilli;Bacillales;Paenibacillaceae;__	0.0313	-57.8179	0.0574	
Firmicutes;Bacilli;Bacillales;Planococcaceae;Planomicrobium	0.0043	2.8601	0.4	
Firmicutes;Bacilli;Bacillales;Staphylococcaceae;Staphylococcus	0.0105	-43.1906	0.284	
Firmicutes;Bacilli;Bacillales;[Exiguobacteraceae];Exiguobacterium	0.0058	-4.8408	0.327	
Firmicutes;Bacilli;Lactobacillales;Aerococcaceae;	0.0368	-498.3021	0.035	†
Firmicutes;Bacilli;Lactobacillales;Carnobacteriaceae;Carnobacterium	0.0185	41.1838	0.0769	
Firmicutes;Bacilli;Lactobacillales;Streptococcaceae;Streptococcus	0.0051	-94.5267	0.399	
Firmicutes;Clostridia;Clostridiales;Clostridiaceae;Clostridium	0.0374	-383.0702	0.0192	†
Firmicutes;Clostridia;Clostridiales;Lachnospiraceae;__	0.0291	-287.0299	0.0514	
Proteobacteria;Alphaproteobacteria;Caulobacterales;Caulobacteraceae;__	0.0427	-28.7123	0.0051	i/n/u
Proteobacteria;Alphaproteobacteria;Caulobacterales;Caulobacteraceae;	0.0056	-27.0532	0.404	
Proteobacteria;Alphaproteobacteria;Caulobacterales;Caulobacteraceae;Brevundimonas	0.0372	-10.3967	0.0158	i
Proteobacteria;Alphaproteobacteria;Caulobacterales;Caulobacteraceae;Caulobacter	0.0197	-284.2794	0.14	
Proteobacteria;Alphaproteobacteria;Caulobacterales;Caulobacteraceae;Mycoplana	0.0001	1.2967	0.917	
Proteobacteria;Alphaproteobacteria;Rhizobiales;__;__	0.019	-71.036	0.0928	
Proteobacteria;Alphaproteobacteria;Rhizobiales;;	0.0066	-166.6442	0.347	
Proteobacteria;Alphaproteobacteria;Rhizobiales;Bradyrhizobiaceae;Bradyrhizobium	0.0737	-215.0021	0.0015	†
Proteobacteria;Alphaproteobacteria;Rhizobiales;Brucellaceae;__	0.0486	15.4689	0.0047	i/n
Proteobacteria;Alphaproteobacteria;Rhizobiales;Brucellaceae;Ochrobactrum	0.0479	36.9233	0.0051	n
Proteobacteria;Alphaproteobacteria;Rhizobiales;Hyphomicrobiaceae;Rhodoplanes	0.0379	-164.944	0.018	†
Proteobacteria;Alphaproteobacteria;Rhizobiales;Methylobacteriaceae;Methylobacterium	0.0215	5.9257	0.0517	
Proteobacteria;Alphaproteobacteria;Rhizobiales;Rhizobiaceae;Agrobacterium	0.0126	-5.7716	0.1455	
Proteobacteria;Alphaproteobacteria;Rhizobiales;Rhizobiaceae;Shinella	0.001	23.7471	0.6878	
Proteobacteria;Alphaproteobacteria;Rhodobacterales;Rhodobacteraceae;Paracoccus	0.0154	-4.7809	0.1056	
Proteobacteria;Alphaproteobacteria;Rhodobacterales;Rhodobacteraceae;Rhodobacter	0	-0.0669	0.986	
Proteobacteria;Alphaproteobacteria;Rhodospirillales;Rhodospirillaceae;	0.0034	-30.3853	0.46	
Proteobacteria;Alphaproteobacteria;Sphingomonadales;Erythrobacteraceae;	0.0376	-8.0289	0.0636	
Proteobacteria;Alphaproteobacteria;Sphingomonadales;Sphingomonadaceae;Blastomonas	0.0017	-14.2161	0.599	
Proteobacteria;Alphaproteobacteria;Sphingomonadales;Sphingomonadaceae;Sphingomonas	0.0017	-3.7418	0.7234	
Proteobacteria;Alphaproteobacteria;Sphingomonadales;Sphingomonadaceae;Sphingopyxis	0.0262	-355.3348	0.0364	†
Proteobacteria;Betaproteobacteria;__;__;__	0.0037	-24.4647	0.464	
Proteobacteria;Betaproteobacteria;Burkholderiales;Alcaligenaceae;	0.0072	-8.6724	0.273	

Proteobacteria;Betaproteobacteria;Burkholderiales;Alcaligenaceae;Achromobacter	0.0277	-71.7073	0.0793	
Proteobacteria;Betaproteobacteria;Burkholderiales;Comamonadaceae;__	0.0004	-0.5966	0.8161	
Proteobacteria;Betaproteobacteria;Burkholderiales;Comamonadaceae;	0.0379	69.3688	0.0147	i/n/u
Proteobacteria;Betaproteobacteria;Burkholderiales;Comamonadaceae;Comamonas	0.0024	-2.9056	0.5916	
Proteobacteria;Betaproteobacteria;Burkholderiales;Comamonadaceae;Hydrogenophaga	0.0001	-3.0539	0.887	
Proteobacteria;Betaproteobacteria;Burkholderiales;Comamonadaceae;Rhodoferax	0.0003	1.6701	0.818	
Proteobacteria;Betaproteobacteria;Burkholderiales;Comamonadaceae;Rubrivivax	0.0073	-21.2496	0.2712	
Proteobacteria;Betaproteobacteria;Burkholderiales;Oxalobacteraceae;__	0.0004	-10.5343	0.8225	
Proteobacteria;Betaproteobacteria;Burkholderiales;Oxalobacteraceae;Janthinobacterium	0.0128	-4.984	0.1403	
Proteobacteria;Betaproteobacteria;Burkholderiales;Oxalobacteraceae;Ralstonia	0.0974	-79.3281	0.0002	i/n/u
Proteobacteria;Gammaproteobacteria;Alteromonadales;Alteromonadaceae;Cellvibrio	0.0139	-55.2975	0.131	
Proteobacteria;Gammaproteobacteria;Enterobacteriales;Enterobacteriaceae;Citrobacter	0.0039	10.9112	0.426	
Proteobacteria;Betaproteobacteria;Rhodocyclales;Rhodocyclaceae;Dechloromonas	0	0.1906	0.976	
Proteobacteria;Gammaproteobacteria;Alteromonadales;[Chromatiaceae];Rheinheimera	0.0667	44.3048	0.0005	†
Proteobacteria;Gammaproteobacteria;Enterobacteriales;Enterobacteriaceae;__	0.0001	4.5854	0.935	
Proteobacteria;Gammaproteobacteria;Enterobacteriales;Enterobacteriaceae;Escherichia	0.0627	-124.5688	0.0047	†
Proteobacteria;Gammaproteobacteria;Pseudomonadales;Moraxellaceae;Acinetobacter	0.0493	-8.2974	0.0036	e/i/n/u
Proteobacteria;Gammaproteobacteria;Pseudomonadales;Moraxellaceae;Enhydrobacter	0.0491	-31.0482	0.013	†
Proteobacteria;Gammaproteobacteria;Pseudomonadales;Moraxellaceae;Psychrobacter	0.0357	-139.0153	0.085	
Proteobacteria;Gammaproteobacteria;Pseudomonadales;Pseudomonadaceae;__	0.0826	20.6634	0.0001	e/i/n/u
Proteobacteria;Gammaproteobacteria;Pseudomonadales;Pseudomonadaceae;Pseudomonas	0.0011	2.5986	0.6977	
Proteobacteria;Gammaproteobacteria;Xanthomonadales;Xanthomonadaceae;__	0.0019	-12.2604	0.577	
Proteobacteria;Gammaproteobacteria;Xanthomonadales;Xanthomonadaceae;Stenotrophomonas	0.007	-3.13	0.2843	
Proteobacteria;Gammaproteobacteria;Xanthomonadales;Xanthomonadaceae;Luteimonas	0.0008	3.3433	0.726	
Proteobacteria;Gammaproteobacteria;Xanthomonadales;Xanthomonadaceae;Thermomonas	0.0024	-5.7038	0.551	
Verrucomicrobia;Verrucomicrobiae;Verrucomicrobiales;Verrucomicrobiaceae;	0.0004	-4.9493	0.809	
TM7;TM7-3;;;	0.0001	-6.4485	0.881	
Verrucomicrobia;Verrucomicrobiae;Verrucomicrobiales;Verrucomicrobiaceae;Akkermansia	0.0048	-29.9354	0.444	
[Thermi];Deinococci;Thermales;Thermaceae;Thermus	0.0009	-13.4704	0.715	

Table S9. Comparisons on bacterial diversity of skin microbiome among the wild populations of five Asia host species and *L. caerulea* before *Bd* inoculation based on linear models and pair test. The linear model included OTU richness or Shannon index as response variable and species as an independent variable. P value based on Bonferroni correction. The red indicates the significance≤0.05.

contrast	estimate	SE	df	t.ratio	p.value
OTU richness					
L. caerulea - B. gargarizans	-33.89	5.22	79	-6.492	<.0001
L. caerulea - D. melanostictus	-10.37	4.75	79	-2.185	0.4776
L. caerulea - P. nigrimaculatus	-17.34	4.1	79	-4.225	0.001
L. caerulea - C. orientalis	-4.03	4.54	79	-0.888	1
L. caerulea - S. raddei	-21.33	4.15	79	-5.141	<.0001
B. gargarizans - D. melanostictus	23.52	5.47	79	4.298	0.0007
B. gargarizans - P. nigrimaculatus	16.55	4.93	79	3.359	0.0181
B. gargarizans - C. orientalis	29.87	5.29	79	5.643	<.0001
B. gargarizans - S. raddei	12.57	4.96	79	2.531	0.2003
D. melanostictus - P. nigrimaculatus	-6.97	4.42	79	-1.577	1
D. melanostictus - C. orientalis	6.34	4.83	79	1.314	1
D. melanostictus - S. raddei	-10.96	4.46	79	-2.455	0.2441
P. nigrimaculatus - C. orientalis	13.32	4.2	79	3.173	0.0322
P. nigrimaculatus - S. raddei	-3.98	3.77	79	-1.056	1
C. orientalis - S. raddei	-17.3	4.24	79	-4.08	0.0016
Shannon index					
L. caerulea - B. gargarizans	-1.7866	0.466	79	-3.831	0.0038
L. caerulea - D. melanostictus	-1.9247	0.424	79	-4.54	0.0003
L. caerulea - P. nigrimaculatus	-1.5979	0.367	79	-4.358	0.0006
L. caerulea - C. orientalis	-1.5659	0.405	79	-3.864	0.0034
L. caerulea - S. raddei	-1.3218	0.371	79	-3.566	0.0093
B. gargarizans - D. melanostictus	-0.1381	0.489	79	-0.282	1
B. gargarizans - P. nigrimaculatus	0.1888	0.44	79	0.429	1
B. gargarizans - C. orientalis	0.2207	0.473	79	0.467	1
B. gargarizans - S. raddei	0.4648	0.443	79	1.048	1
D. melanostictus - P. nigrimaculatus	0.3268	0.395	79	0.827	1
D. melanostictus - C. orientalis	0.3587	0.431	79	0.832	1
D. melanostictus - S. raddei	0.6029	0.399	79	1.512	1
P. nigrimaculatus - C. orientalis	0.0319	0.375	79	0.085	1
P. nigrimaculatus - S. raddei	0.2761	0.337	79	0.819	1
C. orientalis - S. raddei	0.2441	0.379	79	0.645	1

Table S10. PERMANOVA and ANOSIM for Bray-Curtis distance among wild populations of five Asian hosts and *L. caerulea* before *Bd* inoculation. Red indicates the significance≤0.05.

PERMANOVA

Pseudo-F	9.7891
p-value	0.0010

ANOSIM

Group 1	Group 2	Sample size	R	p-value	q-value
B. gargarizans	*C. orientalis*	21	0.7923	0.0010	0.0011
	D. melanostictus	19	0.6505	0.0010	0.0011
	L. caerulea	22	0.9664	0.0010	0.0011
	P. nigromaculatus	28	0.7376	0.0010	0.0011
	S. raddei	27	0.8564	0.0010	0.0011
C. orientalis	*D. melanostictus*	24	0.2124	0.0020	0.0020
	L. caerulea	27	0.9407	0.0010	0.0011
	P. nigromaculatus	33	0.3699	0.0010	0.0011
	S. raddei	32	0.5928	0.0010	0.0011
D. melanostictus	*L. caerulea*	25	0.9352	0.0010	0.0011
	P. nigromaculatus	31	0.4170	0.0010	0.0011
	S. raddei	30	0.5563	0.0010	0.0011
L. caerulea	*P. nigromaculatus*	34	0.9710	0.0010	0.0011
	S. raddei	33	0.9758	0.0010	0.0011
P. nigromaculatus	*S. raddei*	39	0.5956	0.0010	0.0011
